# Topology-adjusted knockout (TAKO): interpretable *in silico* single-gene knockout ranking on wild-type single-cell gene regulatory networks

**DOI:** 10.3389/fbinf.2026.1924703

**Published:** 2026-10-14

**Authors:** Hao Wang, Koji Oba

**Affiliations:** 1 Graduate School of Interdisciplinary Information Studies, The University of Tokyo, Tokyo, Japan; 2 Interfaculty Initiative in Information Studies, The University of Tokyo, Tokyo, Japan

**Keywords:** gene regulatory networks, *in silico* knockout, network propagation, personalised PageRank, single-cell RNA-seq

## Abstract

Predicting single-gene knockout effects from wild-type single-cell RNA-seq data remains challenging when perturbation experiments are costly and existing approaches require knockout-specific model training. Here we introduce *Topology-Adjusted Knockout* (TAKO), a computationally efficient WT-only framework for single-gene KO impact ranking on single-cell gene regulatory networks. The method applies a strict cut-in-and-out intervention on network transition dynamics and quantifies impact by contrasting stationary propagation profiles before and after perturbation under the same restart distribution. In SERGIO simulations, TAKO recovered direct regulator–target structure but did not show a consistent global advantage over a matched absolute Pearson-correlation ranking across network sizes. Direct evaluation against measured responses in *Trem2* and *Nkx2-1* KO data likewise yielded mixed global discrimination, although TAKO contained more differentially expressed genes among the top-ranked candidates in both examples. These results position TAKO as a fast, interpretable topology-based prioritisation method rather than a generally superior predictor of post-KO expression responses.

## 1 Introduction

Elucidating gene function is fundamental for understanding biological processes, disease mechanisms and therapeutic target discovery (Jackson et al., 2018; Plenge et al., 2013). Gene perturbation experiments—especially gene knockout (KO)—are widely regarded as a gold standard for causal functional analysis: by comparing molecular or cellular phenotypes between knockout and wild-type (WT) conditions, one can infer the role of a target gene in a given context (Sha et al., 2014; Wang et al., 2014). Among molecular readouts, transcriptome-wide gene expression provides a rich quantitative phenotype that sensitively reflects KO-induced changes in cell state (Wang et al., 2009).

The advent of single-cell RNA sequencing (scRNA-seq) has moved such analyses from bulk averages to single-cell resolution, enabling the dissection of heterogeneous cell populations and state transitions (Zheng et al., 2017; Luecken and Theis, 2019; Stuart et al., 2019; Hafemeister and Satija, 2019). Combining scRNA-seq with systematic clustered regularly interspaced short palindromic repeats (CRISPR)-based perturbation across many genes and cell types could, in principle, yield a comprehensive atlas of gene function (Dixit et al., 2016; Adamson et al., 2016; Norman et al., 2019; Replogle et al., 2020; Duan et al., 2019). In practice, however, large-scale single-cell perturbation screens remain expensive and technically demanding: genome editing and delivery efficiencies are imperfect, experimental cycles are long, and ethical or safety concerns constrain what can be tested in primary tissues or patient-derived models (Doench, 2018). Moreover, single-cell measurements are noisy and subject to technical variability, which complicates the detection of true differential responses and has motivated dedicated modelling and denoising frameworks for scRNA-seq, including deep count autoencoders and confounder-aware analysis (Squair et al., 2021; Eraslan et al., 2019). Consequently, for many systems we only have WT scRNA-seq, and must anticipate KO effects from WT observations alone. In this WT-only setting, we focus on gene-level KO impact ranking—prioritising KO-responsive targets—rather than reconstructing full post-KO expression profiles.

To bridge this gap, a growing body of work studies *in silico* KO or virtual perturbation: starting from observational gene expression data—often including both WT and perturbed cells—predict the effect of removing or modulating a gene or pathway (Lotfollahi et al., 2019; Wei et al., 2022). Existing *in silico* perturbation methods can be roughly divided into two families, with a related line of network-propagation approaches.

The first family is *graph-centric* and operates directly on WT-derived gene regulatory networks (GRNs). Here one first infers a gene–gene network from WT expression—for example using correlation, regression or co-expression modules—and then scores changes after a virtual perturbation of the graph. A wide range of GRN inference pipelines have been proposed for single-cell data, including integrated workflows such as Single-Cell rEgulatory Network Inference and Clustering (SCENIC), which combine co-expression with motif information to identify regulons and cluster cell states (Aibar et al., 2017). scTenifoldKnk constructs a manifold-aligned single-cell GRN from WT data, perturbs the KO gene in the network, and uses a low-rank embedding to detect differentially regulated targets (Osorio et al., 2022). Other studies benchmark GRN inference algorithms on single-cell transcriptomic data and highlight the challenges posed by sparsity, cellular heterogeneity and uncertain edge directionality. Such methods are attractive because they make explicit use of network topology and can be applied in WT-only regimes, but their performance is tightly coupled to the quality of the inferred GRN—i.e., the accuracy of regulatory edge detection and edge directionality, which remain challenging for sparse scRNA-seq data (Pratapa et al., 2020).

A second family of approaches relies on *representation learning*. These methods train latent generative or graph models on WT data, then emulate interventions in the learned space. For example, scGen uses a variational auto-encoder to model cell states and approximate treatment responses via latent-space arithmetic (Lotfollahi et al., 2019). GenKI learns a variational graph auto-encoder on a WT-derived gene network and simulates the KO of a regulator by editing its latent representation in the inferred graph and propagating the perturbation to its targets (Yang et al., 2023). Related models use latent factors, diffusion-based manifolds or neural network architectures to capture temporal trajectories and, in some cases, underlying regulatory programmes from scRNA-seq data (Trapnell et al., 2014; Haghverdi et al., 2016; van Dijk et al., 2018; Shu et al., 2021). Recent work such as CellOracle further integrates gene regulatory network (GRN) inference with *in silico* gene perturbation to forecast changes in cell identity landscapes at single-cell resolution (Kamimoto et al., 2023). These methods can capture complex, non-linear relationships, but require substantial training and careful hyperparameter tuning, and their KO predictions may be difficult to interpret mechanistically.

Distinct from these generative frameworks, network propagation methods move closer to our setting by leveraging WT-only networks to estimate how a *drug, pathway or gene set* influences different cell populations. Methods in this line construct a gene-, sample- or, in some cases, cell-level network, treat drug targets or pathway genes as “seeds”, and apply diffusion or PageRank-like propagation to score which cells are most sensitive to a given perturbation (Brin and Page, 1998; Andersen et al., 2006; Vanunu et al., 2010; Cowen et al., 2017; Schubert et al., 2018; Guney et al., 2016). For example, approaches inspired by scRank start from WT single-cell data, build a gene-based or cell-based network, and compute influence scores that prioritise cell types likely to respond to a targeted therapy (Li et al., 2024). At the bulk-tissue and protein–protein interaction level, network-based propagation has also been used for *in silico* drug efficacy screening and disease gene prioritisation (Vanunu et al., 2010; Guney et al., 2016). These methods demonstrate that, under a reasonable network, localised propagation can amplify weak genetic signals and improve the detectability of perturbation responses. However, they remain *drug- or pathway-centric*: the output is typically a ranking of cells for a *given* set of seed genes, rather than a gene-level ranking of KO-responsive targets for a single KO gene.

In many practical scenarios, what biologists need is precisely the latter: given only WT scRNA-seq and a candidate KO gene, provide a ranked list of genes that are likely to respond to the KO of that gene. We refer to this as the *WT-only single-gene KO impact ranking* problem: starting from a WT-derived single-cell gene regulatory network (scGRN) and a candidate KO gene, our goal is to assign each gene a topology-based score that serves as a network-theoretic surrogate of its KO responsiveness. This setting differs from (i) manifold-alignment methods such as scTenifoldKnk (Osorio et al., 2022), (ii) representation-learning and variational-graph methods like GenKI that require training global simulators (Yang et al., 2023), and (iii) drug-response propagation methods that assume a set of known targets and output cell-level sensitivity. It calls for a framework that is (a) WT-only, (b) leverages training-free, topology-aware propagation, (c) is explicitly interpretable, and (d) scales to thousands of genes and many candidate KOs.

Here we introduce *Topology-Adjusted Knockout* (TAKO), a computationally efficient WT-only framework for single-gene KO impact ranking on single-cell GRNs. TAKO views a WT-derived scGRN as a weighted directed graph and converts it into a non-negative, row-stochastic transition matrix, thereby defining a Markov chain over genes. To model the knockout of a candidate gene, TAKO applies a strict *cut-in-and-out* intervention directly on this Markov dynamics: it removes all transitions into and out of the KO gene, assigns a pure self-loop to the KO node, and re-normalises affected rows to preserve stochasticity. This yields a topology-adjusted KO transition matrix that matches the intervention semantics used throughout our implementation.

To quantify KO impact, TAKO computes stationary network-propagation equilibria on both the WT and KO transition matrices under the same *uniform restart over non-KO genes*. The resulting KO impact profile is defined as the *difference* between the WT and KO stationary propagation profiles. Depending on downstream use, genes can be ranked by this signed profile, by its positive part, or by its magnitude.

We benchmark TAKO on SERGIO-based simulations with three GRN sizes (100, 400 and 1,200 genes) (Dibaeinia and Sinha, 2020), using simulator-provided directed edges as a structural reference and evaluating area under the receiver operating characteristic curve (AUROC), average precision (AP) and precision–recall curves. We compare against a random ranking, scTenifoldKnk (Osorio et al., 2022), GenKI (Yang et al., 2023), signed Pearson correlation and a matched absolute Pearson-correlation ranking computed from WT cells. We further distinguish pathway-level biological interpretation from direct gene-level evaluation against measured WT–KO expression changes in two public KO datasets. This design allows us to report both the computational advantages and the predictive limitations of TAKO without treating functional enrichment as a substitute for perturbation-response accuracy.

Our contributions are threefold. First, we formalise WT-only single-gene KO ranking as a global topological sensitivity problem on WT-derived scGRNs. Rather than anchoring propagation at the KO gene, we compare WT versus topology-adjusted KO equilibria under a uniform restart to obtain an explicit KO impact profile. Second, we provide an end-to-end pipeline from WT counts to KO impact scores that combines straightforward GRN construction with training-free propagation and scales to large gene sets. Third, we evaluate the method against both a strong naive correlation baseline and measured KO expression changes, thereby identifying where the topology-based ranking is informative and where it does not add stable global predictive value.

## 2 Results

### 2.1 Overview of the TAKO workflow

We first provide an overview of the TAKO workflow from WT single-cell counts to KO impact scores (Figure 1). Given WT scRNA-seq profiles for a cell population, we denote the WT expression matrix by X , with rows as cells and columns as genes. As illustrated in Figure 1A, TAKO first normalises X and constructs a directed WT gene regulatory graph using a PCR-based regression scheme: we compute an SVD-based low-rank representation of X and fit ridge regression in the reduced space to estimate a directed interaction matrix A . We then convert A into a non-negative, row-normalised transition matrix P , which defines a Markov chain over genes.

**FIGURE 1:**
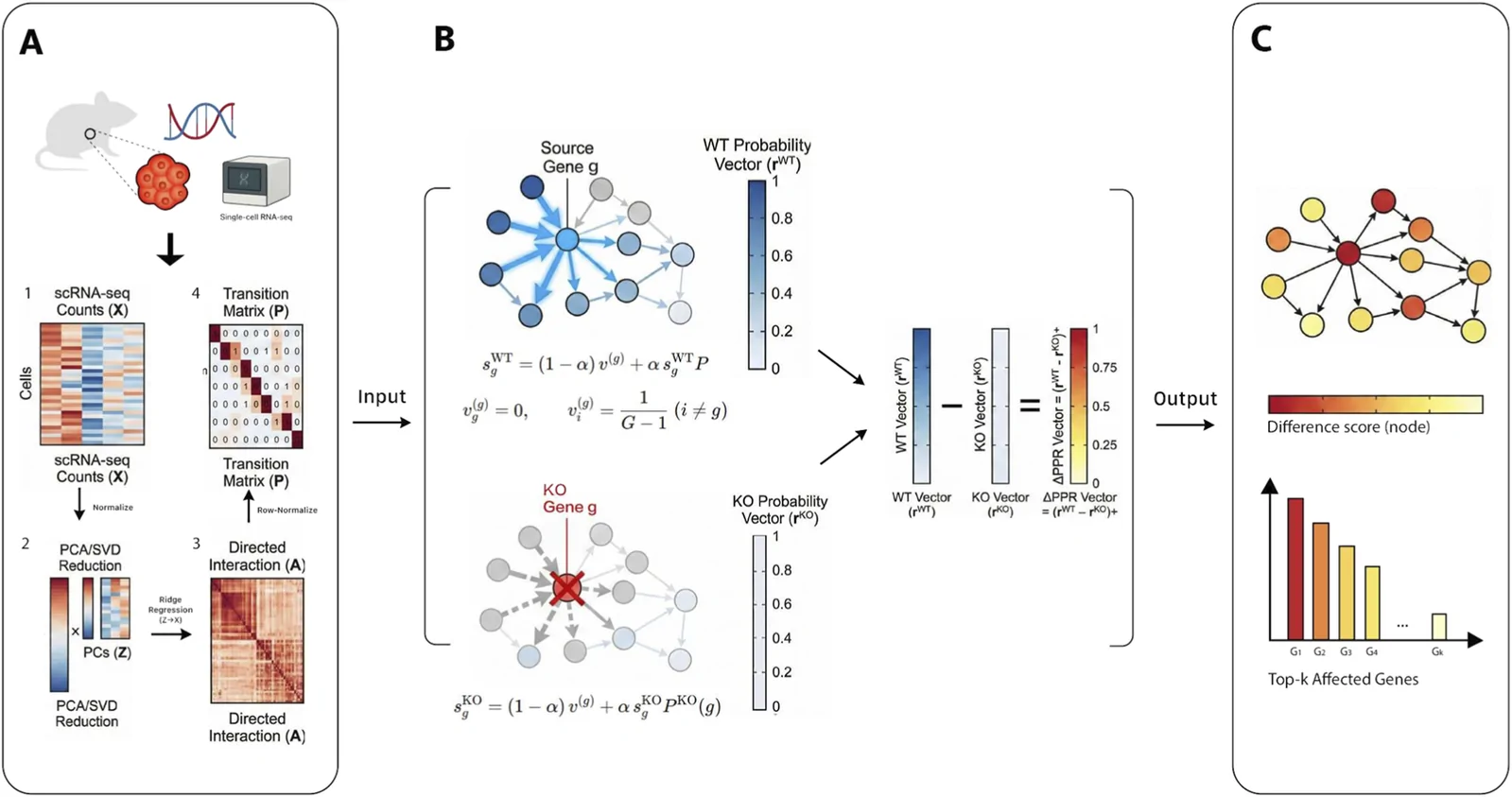
Overview of the TAKO framework. **(A)** From QC’ed wild-type (WT) scRNA-seq counts X , TAKO constructs a WT single-cell gene regulatory network (scGRN) in four steps: (1) normalise WT counts X ; (2) project to a low-dimensional space Z via thin singular value decomposition (SVD); (3) estimate a directed interaction matrix A by batch ridge regression from Z ; (4) convert A into a non-negative, row-normalised transition matrix P defining a Markov chain on genes. **(B)** For a candidate KO gene g , TAKO defines a topology-adjusted KO transition matrix $P_{g}^{\text{KO}}$ by a strict cut-in-and-out (no-in–out) intervention on P : remove all incoming transitions into g (zero the column of g ); remove all outgoing transitions from g (zero the row of g ); assign a pure self-loop at g ; and re-normalise affected rows to preserve stochasticity. **(C)** TAKO quantifies KO impact by contrasting stationary propagation equilibria on the WT and KO graphs under the same restart distribution $v^{\left(g\right)}$ , which is uniform over non- g genes ( $v_{g}^{\left(g\right)}=0$ , $v_{i}^{\left(g\right)}=1/\left(G-1\right)$ for i≠g ). Let $s_{g}^{\text{WT}}$ and $s_{g}^{\text{KO}}$ denote the resulting stationary vectors on P and $P_{g}^{\text{KO}}$ , respectively. The signed KO impact profile (delta_raw) is defined as $\Delta s_{g}^{\text{raw}}=s_{g}^{\text{WT}}-s_{g}^{\text{KO}}$ , with $\Delta s_{g}^{\text{pos}}={\left(\Delta s_{g}^{\text{raw}}\right)}_{+}$ and $\Delta s_{g}^{\text{abs}}=|\Delta s_{g}^{\text{raw}}|$ as alternative derived scores. In this study, quantitative SERGIO evaluation and GSEA use $\Delta s_{g}^{\text{raw}}$ , while direct comparison with unsigned measured KO-response magnitudes uses $\Delta s_{g}^{\text{abs}}$ .

For a candidate KO gene g , TAKO defines a *topology-adjusted* KO by intervening directly on the Markov transition matrix (Figure 1B). Specifically, we apply a strict cut-in-and-out semantic: remove all transitions into g , remove all transitions out of g , assign a pure self-loop at g , and re-normalise affected rows to preserve stochasticity, obtaining a KO transition matrix $P_{g}^{\text{KO}}$ .

To quantify KO impact, TAKO compares stationary propagation equilibria on the WT and KO graphs under the same restart distribution $v^{\left(g\right)}$ , which is uniform over non- g genes ( $v_{g}^{\left(g\right)}=0$ , $v_{i}^{\left(g\right)}=1/\left(G-1\right)$ for i≠g ). Let $s_{g}^{\text{WT}}$ and $s_{g}^{\text{KO}}$ denote the stationary solutions of personalised PageRank (PPR)-style propagation $s=\left(1-\alpha \right)v^{\left(g\right)}+\alpha sP$ on P and $P_{g}^{\text{KO}}$ , respectively (Brin and Page, 1998; Andersen et al., 2006; Cowen et al., 2017). We define the signed KO impact profile as the stationary difference

$$\Delta s_{g}^{\text{raw}}\;=\;s_{g}^{\text{WT}}-s_{g}^{\text{KO}}.$$

Depending on the downstream task, we use the positive part $\Delta s_{g}^{\text{pos}}={\left(\Delta s_{g}^{\text{raw}}\right)}_{+}$ to focus on loss-of-influence, and the magnitude $\Delta s_{g}^{\text{abs}}=|\Delta s_{g}^{\text{raw}}|$ for magnitude-based visualisation. Sorting genes by these KO impact scores yields a ranked list of putative KO-responsive targets for gene g , which we evaluate in simulations, interpret at the pathway level and compare directly with measured KO responses.

### 2.2 Benchmarking TAKO on synthetic SERGIO knockouts

To systematically evaluate whether TAKO can recover local regulator–target structure from WT data alone, we benchmarked TAKO on SERGIO-based simulations with three GRN sizes (100, 400 and 1,200 genes) (Dibaeinia and Sinha, 2020). SERGIO generates scRNA-seq expression data from a known directed GRN under stochastic transcriptional dynamics, enabling controlled evaluation against known structural edges (Figure 2). We considered three SERGIO GRNs with 100, 400 and 1,200 genes. For each KO gene g , simulator-specified outgoing regulatory edges define the set of directly regulated targets used as positive labels. This benchmark therefore evaluates recovery of direct structural neighbours rather than the full transcriptional response following KO.

**FIGURE 2:**
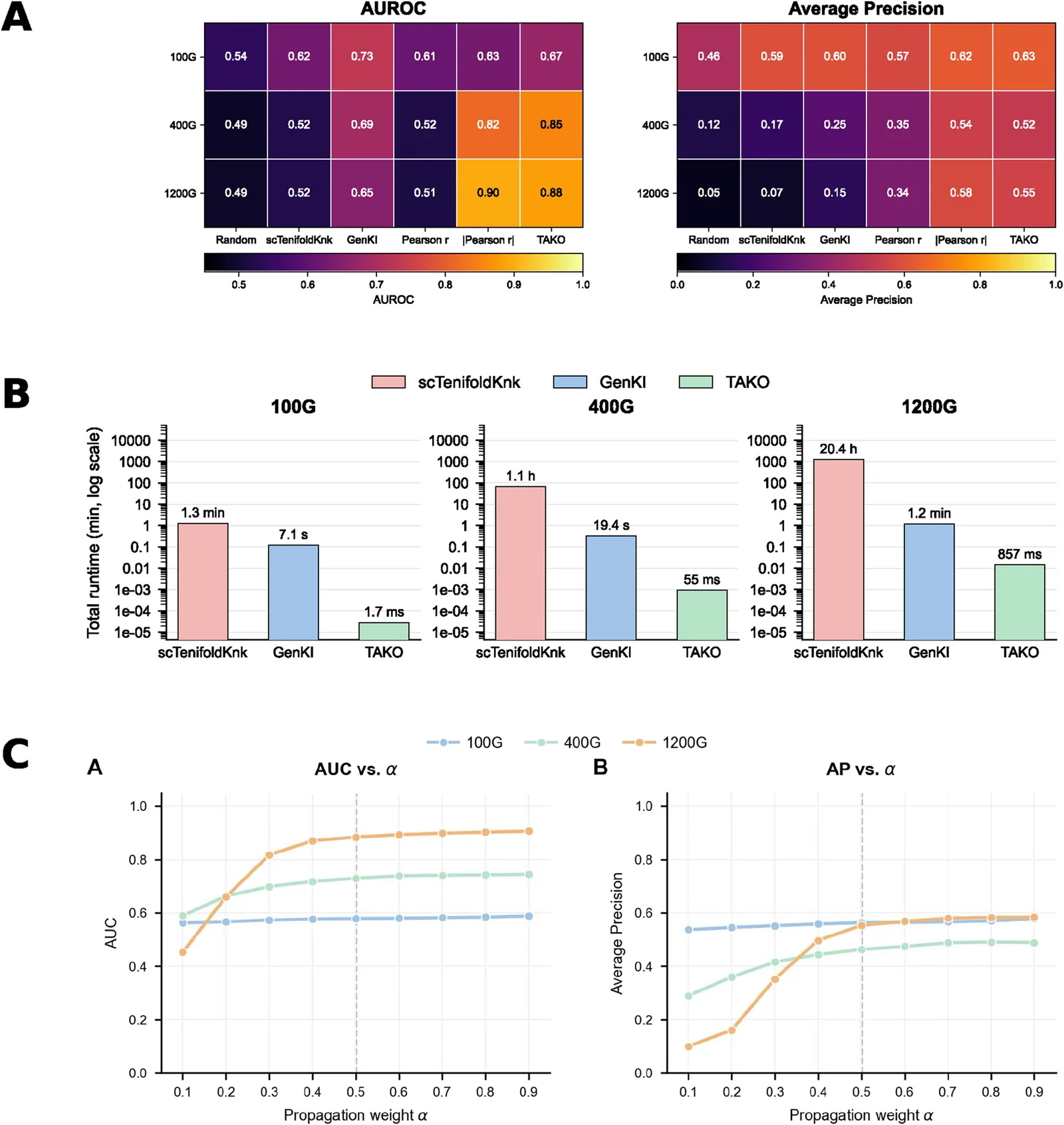
Benchmarking TAKO on synthetic SERGIO knockouts. **(A)** Mean AUROC and average precision (AP) for recovery of direct outgoing simulator edges in the 100-, 400- and 1,200-gene GRNs. Methods comprise a fixed-seed random ranking, scTenifoldKnk, GenKI, signed Pearson correlation, absolute Pearson correlation and TAKO. Accuracy values use prespecified KO lists defined by simulator-GRN out-degree >10 , comprising 6, 25 and 45 KO genes, respectively. Because the reference labels encode adjacency rather than the direction of a post-KO response, absolute Pearson correlation is the primary naive comparator; signed correlation is shown to make this choice explicit. **(B)** Cumulative recorded runtime (log scale) for the same prespecified KO lists of 6, 25 and 45 genes. Recorded per-KO runtimes were summed, and for GenKI one WT model-training time was added to the subsequent KO-scoring times; TAKO and scTenifoldKnk are training-free and include only their recorded per-KO propagation/inference times. **(C)** Sensitivity of AUROC and AP to the PageRank continuation probability α under the uniform-restart impact definition used by TAKO.

Candidate KO genes were defined by simulator-GRN out-degree >10 , giving 6, 25 and 45 KOs in the three networks. The same prespecified KO lists were used for the revised accuracy comparison. We compared TAKO against a random ranking, scTenifoldKnk (Osorio et al., 2022), GenKI (Yang et al., 2023), signed Pearson correlation and absolute Pearson correlation. All methods used WT counts or WT-derived graphs only; ground-truth edges were used solely for evaluation. For each KO gene, we ranked candidate genes j≠g by the configured TAKO impact score $\Delta s_{g,j}$ under the selected delta-mode and computed AUROC and AP, then summarised performance across all KOs within each network size. In the SERGIO experiments reported here, we used delta-mode=raw, i.e., ranking by $\Delta s_{g,j}^{\text{raw}}$ .

Against scTenifoldKnk and GenKI, TAKO showed competitive discrimination of simulator-defined direct targets in the 400- and 1,200-gene settings, whereas GenKI had the highest mean AUROC in the 100-gene setting (Figure 2A). The matched absolute-correlation comparison provided a more stringent reference. For the 100-gene network, mean AUROC was 0.674 for TAKO and 0.630 for absolute correlation, while mean AP was 0.627 and 0.624, respectively; with only six KO targets, the paired 95% confidence intervals were wide and included zero. For the 400-gene network, TAKO had higher mean AUROC (0.853 versus 0.816; mean paired difference 0.036, bootstrap 95% CI 0.010–0.063), whereas mean AP was slightly lower (0.521 versus 0.543; difference −0.021 , 95% CI −0.072 to 0.028). For the 1,200-gene network, absolute correlation was slightly higher in both AUROC (0.897 versus 0.884; difference −0.013 , 95% CI −0.037 to 0.011) and AP (0.580 versus 0.553; difference −0.027 , 95% CI −0.062 to 0.009). Thus, TAKO recovered substantial structural signal but did not provide a consistent global improvement over the naive correlation ranking. Precision–recall curves for TAKO, signed and absolute Pearson correlation, and a fixed-seed random ranking are provided in Supplementary Figure S7.

Signed Pearson correlation was substantially weaker than absolute correlation in the 400- and 1,200-gene networks (AUROC 0.518 and 0.513 versus 0.816 and 0.897; AP 0.346 and 0.340 versus 0.543 and 0.580, respectively). This difference is expected because the structural reference records whether an outgoing edge exists but does not label whether the target should increase or decrease after KO. Reporting only signed correlation would therefore understate the strength of the naive association baseline for this evaluation task.

The Spearman rank-correlation sensitivity analysis reproduced the same pattern. For the 100-gene network, signed and absolute Spearman correlation gave mean AUROC/AP values of 0.615/0.570 and 0.618/0.600, respectively. The corresponding values were 0.520/0.356 and 0.819/0.555 for the 400-gene network, and 0.514/0.338 and 0.897/0.580 for the 1,200-gene network. Thus, in the 400- and 1,200-gene networks, absolute Spearman correlation was substantially stronger than signed Spearman correlation and closely matched absolute Pearson correlation. The advantage of absolute correlation in this structural-reference task is therefore not dependent on the linear scale of Pearson correlation (Supplementary Table S6).

The 100-gene result requires additional caution. Only six genes passed the prespecified out-degree filter, and the mean positive prevalence was 0.419 compared with 0.111 and 0.043 in the 400- and 1,200-gene settings, respectively. This produces a high random AP baseline and substantial between-target uncertainty; apparent differences in the smallest network should therefore not be interpreted as evidence of a scale-related advantage.

In addition to accuracy, TAKO is computationally efficient. Each KO requires only PageRank-style stationary propagation on sparse matrices (WT and topology-adjusted KO), with no KO-specific model training. Accordingly, the configured KO scans completed within seconds on standard hardware, whereas GenKI was slower because of model training and scTenifoldKnk was substantially slower in our implementation (Figure 2B). Because implementations and workloads differ, this panel describes computational scale rather than a hardware-normalised algorithmic comparison. Absolute correlation is computationally simpler than all network-based methods and is therefore not used to support a speed advantage for TAKO.

Finally, we assessed sensitivity to the continuation probability α (Figure 2C). Performance improves when increasing α from very small values to around 0.4–0.5 and then remains broadly stable. We therefore fixed α=0.5 for subsequent experiments.

We further examined robustness to retained edge fraction, GRN construction and the operational definition of knockout on the 400-gene benchmark (Supplementary Figure S6). Performance depended on both network density and intervention choice. The default PCR graph outperformed propagation on a matched-density absolute-correlation graph, while strict cut-in-and-out and row redistribution gave similar performance and cutting outgoing edges alone was weaker. These one-factor-at-a-time analyses support the use of the reported default configuration but do not establish that it is universally optimal.

### 2.3 Pathway-level interpretation on real single-cell scRNA-seq datasets

Having established that TAKO can recover ground-truth regulator–target edges in simulations, we next asked whether its KO rankings are biologically meaningful in real single-cell datasets. We collected four public scRNA-seq datasets centred on biologically important regulators in defined cell populations: *Trem2* KO in mouse microglia, *Nkx2-1* KO in mouse AT1 epithelial cells, a human airway epithelial dataset in COVID-19 analysed through virtual KO of *STAT1*, and a mouse neuronal dataset probing *Mecp2* deficiency (Figures 3, 4).

**FIGURE 3:**
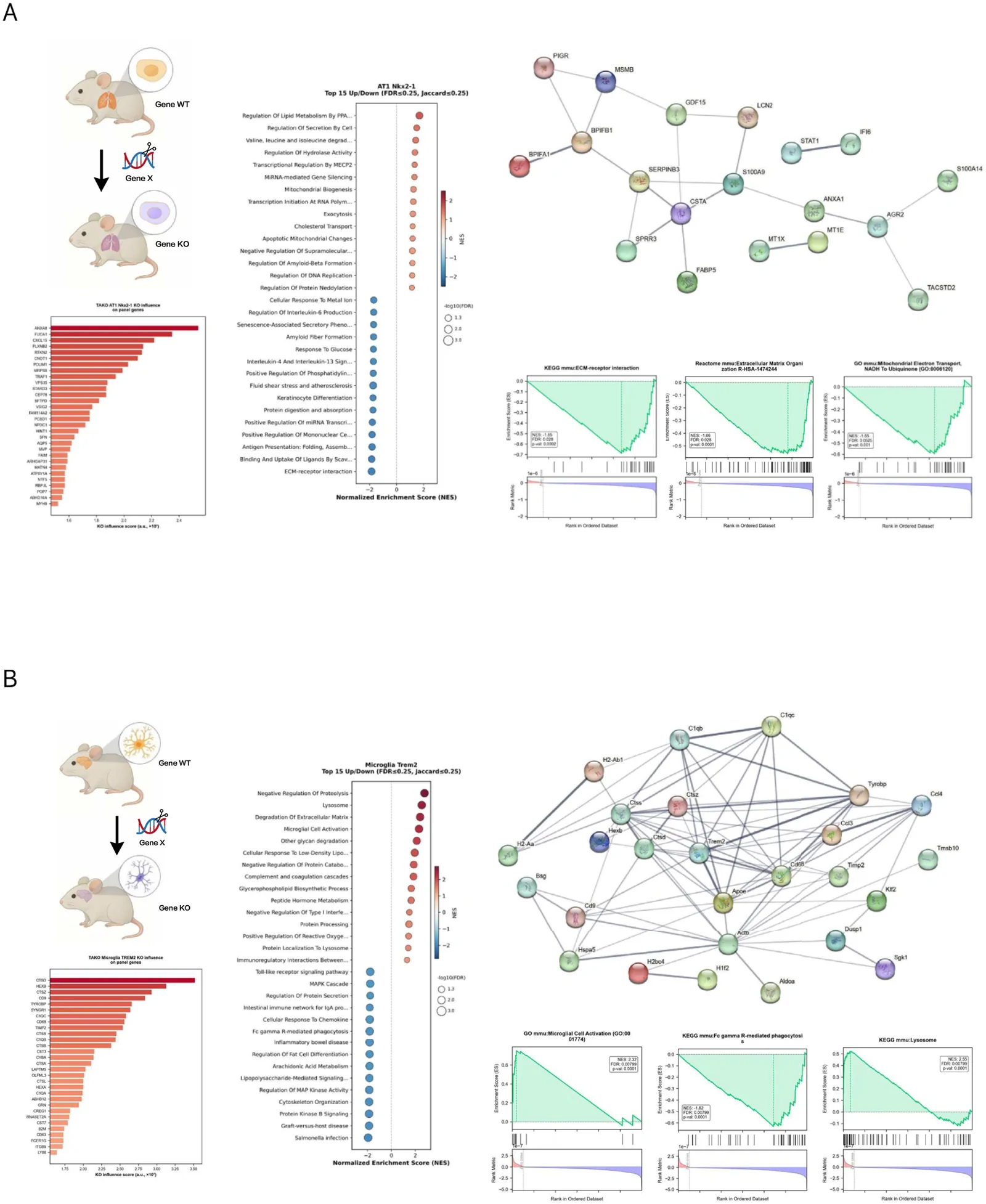
Pathway-level enrichment of TAKO KO impact profiles in two mouse KO datasets. Dot plots of preranked GSEA results using $\Delta s_{g}^{\text{raw}}$ as the rank metric for **(A)** *Trem2* knockout in mouse microglia and **(B)** *Nkx2-1* knockout in mouse AT1 epithelial cells. Displayed terms are selected *algorithmically* for visualisation by the fixed rule: FDR≤0.25 , Top-15 per direction, and leading-edge Jaccard ≤0.25 . Dot colour encodes NES. Dot size is proportional to $-\log_{10}\left(FDR\right)$ with a fixed visual cap for cross-panel comparability (values above 3, i.e., $FDR<10^{-3}$ , are clipped).

**FIGURE 4:**
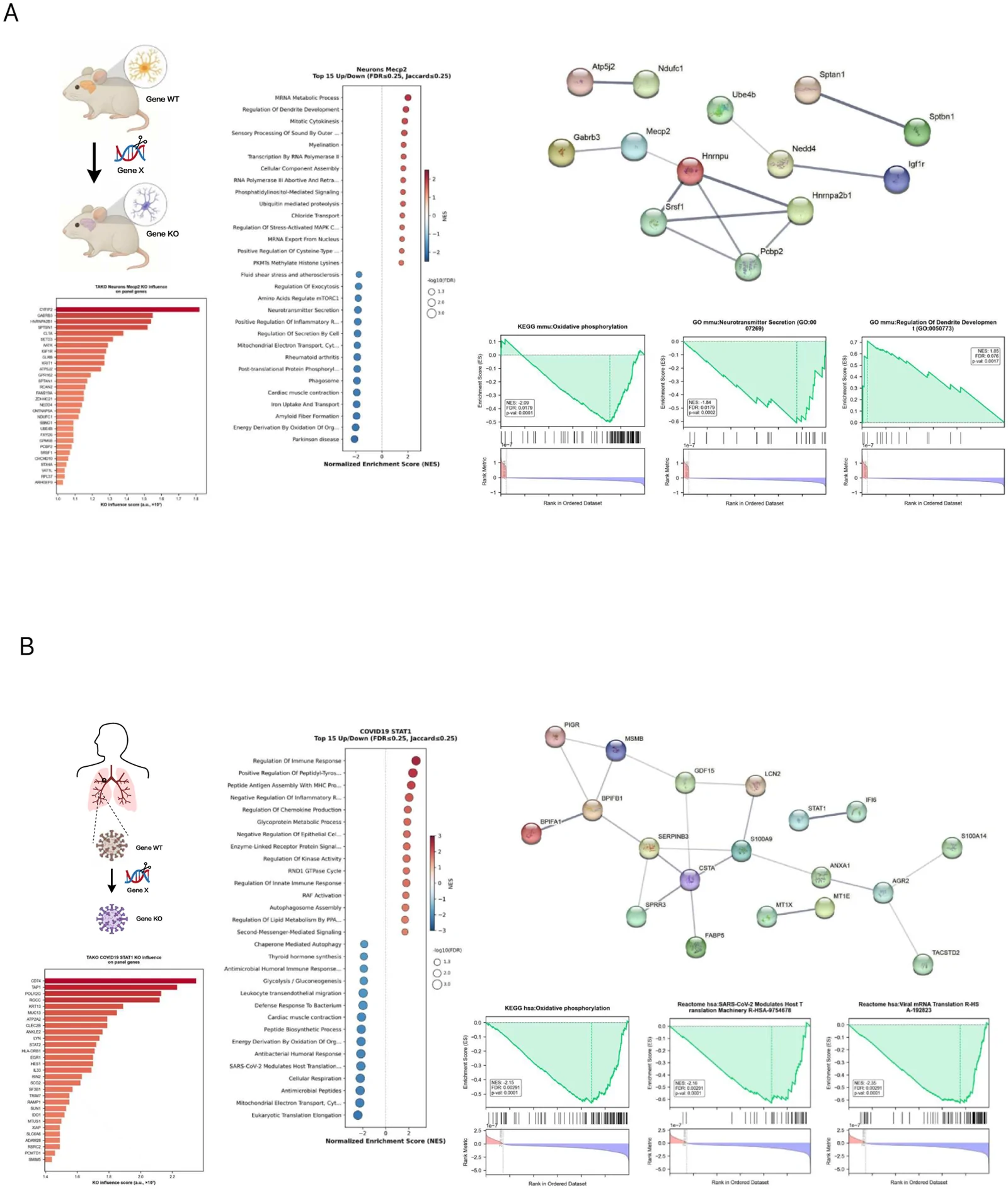
Pathway-level enrichment of TAKO KO impact profiles in human epithelial and mouse neuronal single-cell datasets. Dot plots of preranked GSEA results using $\Delta s_{g}^{\text{raw}}$ as the rank metric for **(A)** Mecp2 deficiency in mouse neurons and **(B)** virtual KO analysis of STAT1 in human airway epithelial cells in COVID-19. Displayed terms are selected using the same fixed rule as Figure 3: FDR≤0.25 , Top-15 per direction, and leading-edge Jaccard ≤0.25 . Dot colour encodes NES, and dot size encodes $-\log_{10}\left(FDR\right)$ with the same fixed cap for comparability.

For each dataset, we ran TAKO on WT cells only, computed the signed KO impact profile $\Delta s_{g}^{\text{raw}}$ for the corresponding KO gene g , and used $\Delta s_{g}^{\text{raw}}$ as the rank metric in preranked gene set enrichment analysis (GSEA) (Subramanian et al., 2005). Gene-set collections from the Gene Ontology (GO) (Ashburner et al., 2000), Kyoto Encyclopedia of Genes and Genomes (Kanehisa and Goto, 2000) and Reactome (Jassal et al., 2020) were used with species-appropriate annotations, and enrichment was summarised by normalised enrichment score (NES) and false discovery rate (FDR)-adjusted p -values (Benjamini and Hochberg, 1995).

#### 2.3.1 Dot-plot selection logic

For visualisation only, we applied the following fixed rule to the full GSEA output for each KO: (i) filter gene sets with FDR≤0.25 ; (ii) sort remaining terms by |NES| (descending), then by smaller FDR, then by term name; (iii) select up to Top-15 positively enriched terms (NES>0) and Top-15 negatively enriched terms (NES<0) *separately*; (iv) within each direction, perform term-level deduplication using Jaccard similarity of leading-edge gene sets, keeping a candidate term only if its leading-edge overlap with every already-selected term in the same direction satisfies Jaccard≤0.25 . This selection affects display only; all enrichment statistics are computed on the full tested gene-set collection.

In the microglial KO dataset, where the lipid-sensing receptor *Trem2* is knocked out in mouse microglia (Nugent et al., 2020), enriched pathways include lysosomal organisation, phagosome-related processes and complement activation. These processes align with reported consequences of *Trem2* perturbation and microglial activation programs (Shi and Holtzman, 2018).

In the AT1 epithelial dataset, *Nkx2-1* disruption is associated with pathways related to extracellular matrix organisation, cell adhesion and epithelial differentiation, consistent with the role of *Nkx2-1* in maintaining AT1 identity and alveolar barrier properties (Little et al., 2019).

In the human airway epithelial context, using the TAKO-derived ranking obtained from virtual KO analysis of the interferon-signalling regulator *STAT1*, we observe enrichment for interferon signalling, antigen processing/presentation and antiviral response pathways, consistent with reported epithelial interferon programs in COVID-19 airway single-cell studies (Chua et al., 2020).

In the neuronal dataset, TAKO KO impact profiles for *Mecp2* deficiency are enriched for synaptic vesicle cycling, axon and cytoskeleton-related processes, consistent with established roles of MeCP2 in neuronal maturation and Rett syndrome biology (Nan et al., 1997; Lyst and Bird, 2015).

Taken together, these applications show that TAKO yields WT-only KO impact profiles whose ranked genes aggregate into pathway-level signatures concordant with known biology across tissues, species and perturbation contexts. This supports biological plausibility, but pathway enrichment alone does not establish agreement with measured post-KO expression changes.

### 2.4 Evaluation against measured KO responses

We next used measured KO cells only as an external reference while keeping both TAKO and the correlation baseline strictly WT-only. For *Trem2*, the analysis contained 765 WT and 885 KO microglial cells; 201 of 2,999 evaluated non-KO genes met the prespecified differential-expression threshold. The absolute-correlation ranking had higher global discrimination than TAKO (AUROC 0.636 versus 0.571; AP 0.108 versus 0.098; Figures 5A,B), although TAKO contained more reference-positive genes near the head of the list (P@10/P@20/P@50: 0.20/0.20/0.22 for TAKO versus 0.00/0.05/0.10 for correlation; Figure 5C).

**FIGURE 5:**
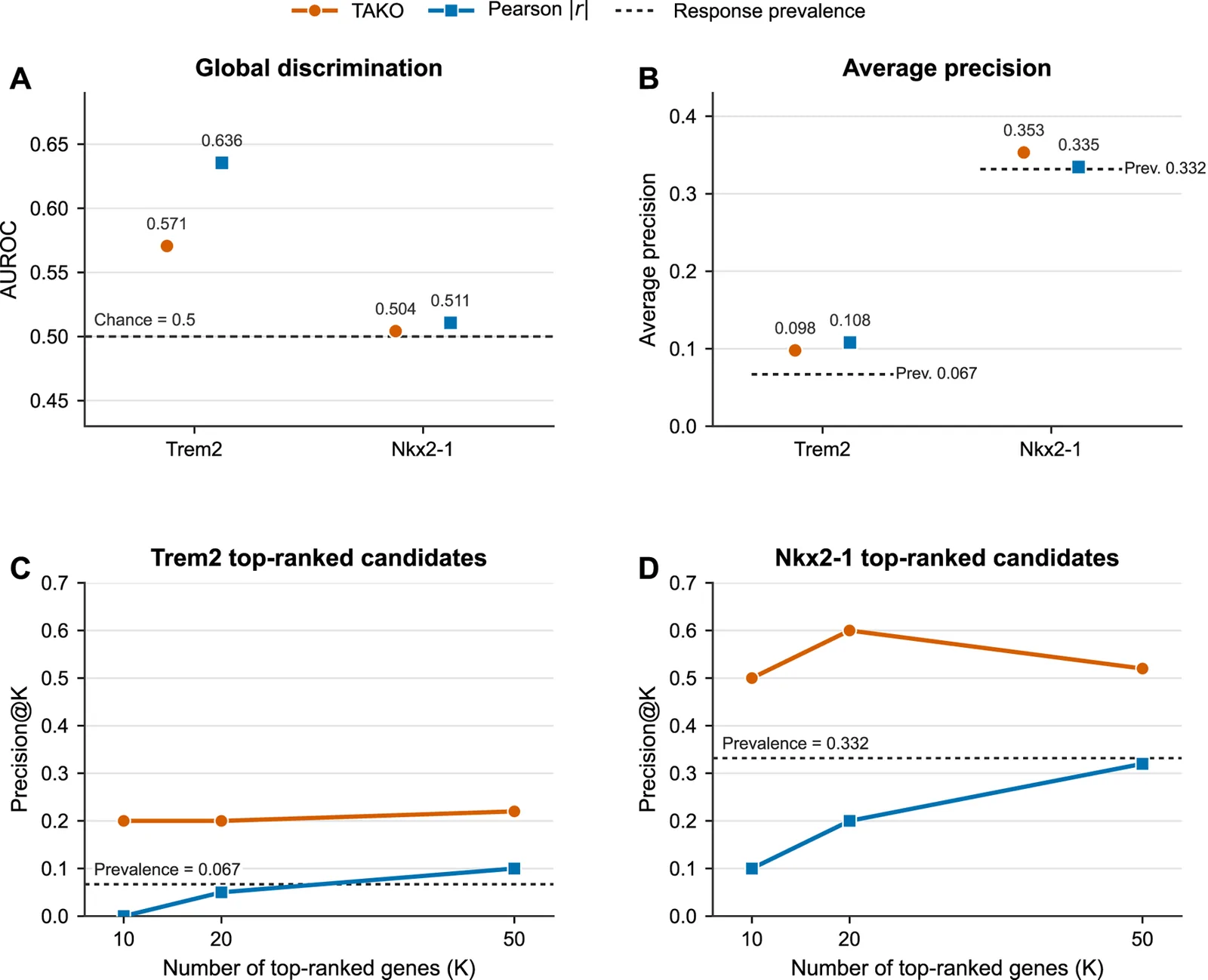
Direct evaluation against measured knockout-induced expression changes. Both TAKO and the absolute Pearson-correlation baseline use WT cells only; KO cells are withheld from prediction and used solely to define the external differential-expression reference. **(A)** AUROC for agreement with the measured-response reference. The dashed line marks chance AUROC (0.5). **(B)** Average precision (AP). Short dashed segments mark the prevalence of reference-positive genes, corresponding to the expected AP of an uninformative ranking. **(C,D)** Precision among the top 10, 20 and 50 candidates for *Trem2* KO in microglia and *Nkx2-1* KO in the corrected AT1 subset, respectively. Horizontal dashed lines mark response prevalence. The KO gene itself is excluded. Because each dataset contains one KO target, the Top- k comparisons are descriptive and do not support across-target statistical inference.

For the corrected AT1 subset of the *Nkx2-1* dataset, the analysis contained 465 WT and 159 KO cells, with 995 reference-positive genes among 2,999 evaluated non-KO genes. Both methods had AUROC close to random (0.504 for TAKO and 0.511 for correlation), while AP was 0.353 and 0.335, respectively (Figures 5A,B). The corresponding P@10/P@20/P@50 values were 0.50/0.60/0.52 for TAKO and 0.10/0.20/0.32 for correlation (Figure 5D).

These two examples do not establish a general Top- k advantage: each dataset contributes only one KO target, and the cell-level differential-expression reference does not provide replicate-aware inference across independent animals. We therefore report the Top- k pattern descriptively and conclude that the available real-data evidence is mixed rather than consistently favourable to TAKO.

## 3 Discussion

In this work we introduced TAKO, a lightweight framework that requires no KO-specific model training for *in silico* single-gene KO ranking on wild-type single-cell gene regulatory networks. Starting from WT scRNA-seq profiles, TAKO constructs a directed, row-stochastic gene–gene transition matrix and defines knockout as a structural cut-in-and-out intervention on the induced Markov dynamics. The difference between WT and KO stationary propagation equilibria yields an explicit topology-based sensitivity profile rather than a reconstructed post-KO expression profile. The revised analyses clarify both what this profile captures and what it does not.

SERGIO supplies a known directed GRN, and our positive labels are the simulator-defined direct outgoing neighbours of each KO gene. This is a valid controlled benchmark of structural neighbourhood recovery, consistent with the evaluation design used in prior WT-only virtual-KO studies, including GenKI, but it is not a direct simulation of the complete transcriptomic response after gene deletion. Indirect, compensatory and cell-state-dependent expression responses may not coincide with direct GRN edges. The SERGIO result therefore supports the narrower conclusion that TAKO recovers part of a known local network structure from WT expression; it cannot by itself establish accurate prediction of experimental post-KO fold changes.

The addition of absolute Pearson correlation further narrows the predictive claim. The SERGIO positives are direct downstream neighbours of the KO gene, but the labels do not distinguish activating from inhibitory regulation. Signed Pearson correlation consequently ranks strongly negatively correlated true downstream genes near the bottom, whereas absolute Pearson correlation ranks both strongly positive and strongly negative associations near the top. The same signed-versus-absolute pattern was reproduced by Spearman correlation, showing that this result is not specific to Pearson’s linear scale. Thus, the strong performance of absolute correlation reflects a particularly close match between an unsigned association score and the unsigned structural-reference task.

TAKO is also compatible with this task because removing both incoming and outgoing edges of the KO gene can produce large local propagation changes among its direct neighbours. By contrast, GenKI and scTenifoldKnk have more complex modelling objectives and are not designed solely for simple direct-neighbour recovery; GRN inference, model training and subsequent propagation or embedding may attenuate local co-variation signals that are directly rewarded by the present labels. These results should therefore not be interpreted as showing that other methods are generally inferior. Correlation was a strong baseline in all three SERGIO networks, and TAKO did not improve both AUROC and AP consistently across network sizes. The value of TAKO is instead its fast, interpretable and WT-only topology-based ranking, rather than comprehensive superiority over correlation.

The real-data analyses lead to a similarly cautious interpretation. Pathway enrichment establishes that the ranked genes form biologically coherent modules, but network-derived rankings are expected to favour co-regulated modules and enrichment cannot substitute for direct accuracy evaluation. Against measured differential-expression references, *Trem2* favoured correlation globally, whereas both methods were close to random by AUROC for *Nkx2-1*; TAKO showed higher descriptive Top- k precision in both examples. Because only two KO targets were available and the present analysis did not provide replicate-aware inference across independent animals, these Top- k observations are hypothesis-generating rather than evidence of a general advantage.

Several limitations follow from the formulation. First, TAKO inherits errors and biases from the inferred GRN, including uncertainty in edge direction and sensitivity to gene selection, dimensionality reduction and sparsification. Second, conversion to non-negative transition weights discards the sign of regulatory interactions; the present scores should not be interpreted as predicting the direction or magnitude of expression change. Third, the strict cut-in-and-out intervention is a network surrogate rather than a mechanistic model of transcriptional compensation, incomplete knockout or cell-state adaptation. Fourth, the current implementation addresses one KO at a time and does not model combinatorial perturbations. Finally, the high positive prevalence and small number of eligible targets in the 100-gene SERGIO network limit cross-scale interpretation.

Within these boundaries, TAKO remains useful as a fast and auditable screening procedure when only WT data are available. It can provide a topology-derived candidate list alongside a correlation ranking, allowing investigators to identify consensus and complementary candidates before experimental follow-up. Future work should evaluate this use in multi-target Perturb-seq datasets with independent biological replication and prespecified Top- k endpoints, and should determine whether alternative or signed GRNs yield reproducible gains over simple co-variation.

## 4 Methods

### 4.1 Preprocessing of single-cell expression data

All prediction analyses start from quality-controlled wild-type single-cell RNA-seq count matrices; measured KO cells are used only to construct the external response reference in the direct validation analyses described below. For each dataset, we first standardised matrix orientation based on gene and cell identifier conventions and then oriented the matrix consistently for downstream processing. Following standard scRNA-seq workflows (Luecken and Theis, 2019; Hafemeister and Satija, 2019; Wolf et al., 2018), we perform library-size normalisation and log-transformation:

$$X_{ij}^{\text{norm}}=\log\;\left(1+10^{4}\frac{X_{ij}}{L_{i}}\right),$$

where $L_{i}=\sum_{j}X_{ij}$ is the total count of cell i .

To focus on informative genes, we select highly variable genes (HVGs) by variance in the log-normalised space and retain the top H genes. When evaluating multiple KOs on the same dataset, we ensure KO genes are retained by taking

$$G={HVG}_{H}\cup K,$$

where K is the KO candidate set or a user-provided force-include list. For SERGIO simulated datasets, genes with total expression counts below 60 were filtered out prior to normalisation.

### 4.2 Construction of WT gene regulatory graphs

We represent the WT single-cell gene regulatory network as a directed weighted graph over genes, with transition matrix $P\in R^{G\times G}$ used for random-walk propagation.

#### 4.2.1 PCR-based directed graph

In our primary configuration, we construct the transition matrix P using principal component regression (PCR). From the log-normalised matrix restricted to G , we transpose the data to $X^{⊤}\in R^{G\times C}$ (genes × cells), mean-center each gene across cells, and compute a thin SVD:

$$X_{c}=USV^{⊤}.$$

Keeping the top D components, let $U_{D}\in R^{G\times D}$ , $S_{D}\in R^{D}$ , $V_{D}\in R^{C\times D}$ , and define low-dimensional cell features $Z=V_{D}\text{diag }\left(S_{D}\right)\in R^{C\times D}$ . We solve ridge regression in batch:

$$\beta ={\left(Z^{⊤}Z+\lambda I\right)}^{-1}Z^{⊤}X_{c}^{⊤},$$

with ridge penalty λ , then back-project to obtain directed coefficients:

$$A=U_{D}\beta \in R^{G\times G},\;diag\left(A\right)=0.$$

To sparsify, we keep the top p% off-diagonal entries by $\left|A_{ij}\right|$ . All remaining entries are set to zero. We then define non-negative edge weights

$$W=\left|A_{\text{masked}}\right|,$$

and apply row normalisation to obtain P .

### 4.3 Topology-adjusted knockout and personalised PageRank

#### 4.3.1 Definition of topology-adjusted KO

For a KO gene g with index $i_{g}$ , we use strict cut-in-and-out semantics (no-in-out):

1. Remove incoming edges by setting column $i_{g}$ to zero: $P_{ui_{g}}\leftarrow 0,\;\forall u$ .
2. Remove outgoing edges by setting row $i_{g}$ to zero and then enforcing a self-loop: $P_{i_{g}v}\leftarrow 0,\;\forall v$ , and $P_{i_{g}i_{g}}\leftarrow 1$ .
3. Re-normalise rows, and replace any zero-sum row with a self-loop.

This yields the KO transition matrix $P_{g}^{\text{KO}}$ , which blocks both inflow into and outflow from g while preserving valid transition dynamics.

#### 4.3.2 Personalised PageRank and KO impact scores

Given transition matrix P , restart distribution $v\in R^{G}$ with $\sum_{i}v_{i}=1$ , and continuation probability α∈(0,1) , we compute the stationary row vector s by

$$s=\left(1-\alpha \right)v+\alpha sP,$$

using fixed-point iteration until $\ell_{1}$ -convergence with tolerance $10^{-8}$ and maximum 200 iterations.

For each KO gene g , the experiments reported here use a uniform restart over non-KO genes:

$$v_{i}^{\left(g\right)}=\left\{\begin{array}{cc} 0, & i=i_{g},\\ \frac{1}{G-1}, & i\neq i_{g}. \end{array}\right.$$

We compute $s_{g}^{\text{WT}}$ on P and $s_{g}^{\text{KO}}$ on $P_{g}^{\text{KO}}$ with the same $v^{\left(g\right)}$ .

The KO impact profile is defined by

$$\Delta s_{g,j}^{\text{raw}}=s_{g,j}^{\text{WT}}-s_{g,j}^{\text{KO}},$$

with derived variants

$$\Delta s_{g,j}^{\text{pos}}=\max\left\{\Delta s_{g,j}^{\text{raw}},0\right\},\;\Delta s_{g,j}^{\text{abs}}=|\Delta s_{g,j}^{\text{raw}}|.$$

We refer to $\Delta s_{g}$ as the KO impact profile and $\Delta s_{g,j}$ as the KO impact score. In the reported analyses, raw differential scoring is used for SERGIO and GSEA, whereas absolute differential scoring is used for direct comparison with unsigned measured-response magnitudes.

### 4.4 SERGIO simulations and evaluation metrics

For benchmarking, we use SERGIO-simulated single-cell RNA-seq datasets with known directed GRNs at multiple network sizes. The simulator-provided GRN is binarised to obtain true regulator-target edges. Candidate KO genes are preselected from the simulator GRN by strict outgoing degree $\deg^{\mathrm{out}}\left(g\right)>10$ , and the resulting list is provided as the KO sequence file. This gives 6, 25 and 45 KO genes in the 100-, 400- and 1,200-gene networks, respectively.

For each KO gene g , the positive set is the outgoing true targets:

$$P_{g}=\left\{j\neq g:\;A_{gj}^{\text{true}}=1\right\}.$$

For each KO, we score all j≠g by the selected Δs mode and compute:

- AUROC.
- AP (Average Precision).
- The full precision–recall curve.

We macro-average per-KO metrics across eligible KOs. For the four methods with matched per-gene score vectors—TAKO, signed Pearson correlation, absolute Pearson correlation, and a fixed-seed random ranking—per-KO precision–recall curves were mapped to a common recall grid using a monotone precision envelope and then macro-averaged within each network size. Pointwise 95% confidence bands were calculated from 1,000 fixed-seed bootstrap resamples of KO genes, and the mean structural-positive prevalence was displayed as the random-reference level (Supplementary Figure S7).

#### 4.4.1 Pearson-correlation baselines

For every KO gene g , we compute the Pearson correlation between its log-normalised WT expression vector and that of each candidate gene j≠g . Candidates are ranked either by signed correlation $r_{g,j}=\text{cor }\left(X_{g},X_{j}\right)$ or by absolute correlation $r_{g,j}^{\text{abs}}=|\text{cor }\left(X_{g},X_{j}\right)|$ . Both baselines use exactly the same WT cells, candidate genes, KO targets and structural reference labels as TAKO. Absolute correlation is the primary naive comparator because the SERGIO edge labels encode adjacency but do not encode the direction of the post-KO expression response; signed correlation is retained to show the effect of this evaluation choice.

#### 4.4.2 Spearman-correlation sensitivity analysis

For every KO gene g , Spearman correlation is computed by replacing each gene’s log-normalised WT expression values with their ranks and then calculating the Pearson correlation between rank vectors; tied values are assigned their average rank. Candidates are ranked either by signed Spearman correlation $\rho_{g,j}$ or by its absolute value $\left|\rho_{g,j}\right|$ . The Spearman baselines use the same WT cells, candidate genes, KO targets and structural reference labels as the Pearson baselines and TAKO. Spearman correlation is included as a sensitivity analysis, while Pearson correlation remains the primary correlation baseline.

### 4.5 Measured WT–KO response evaluation

We performed direct gene-level evaluation in the scRNA-seq subseries GSE130626 (part of GSE130627) for *Trem2* and GSE129584 (part of GSE129628) for *Nkx2-1*. The *Nkx2-1* analysis was restricted to the corrected AT1-cell subset. For each dataset, TAKO and the absolute-correlation baseline were computed using WT cells only; KO cells were withheld from prediction and used solely to construct the external response reference.

WT and KO counts were independently normalised to counts per 10,000 and log-transformed. For each gene, we applied a two-sided Wilcoxon rank-sum test to cell-level expression values, adjusted across genes by the Benjamini–Hochberg procedure, and defined a reference-positive gene by FDR ≤0.05 and absolute ${\text{log}}_{2}$ fold change ≥0.25 . The KO gene itself was excluded from evaluation. For measured-response evaluation, TAKO candidates were ranked by $\left|\Delta s_{g,j}^{\text{raw}}\right|$ , because the non-negative transition network is not designed to predict the sign of an expression change. We evaluated agreement using AUROC, AP, and precision at k∈{10,20,50} , as reported in the main-text figure.

Cells are not independent biological replicates. The differential-expression labels are therefore used as a descriptive external ranking reference and not as replicate-aware causal inference.

### 4.6 Sensitivity and ablation analyses

We conducted a one-factor-at-a-time sensitivity analysis on the 400-gene SERGIO benchmark using the same 25 prespecified KO genes as in the primary comparison. Starting from the default configuration (PCR graph, 15% retained off-diagonal edges, α=0.5 , strict cut-in-and-out intervention), we varied: (i) propagation weight α from 0.1 to 0.9 in increments of 0.1; (ii) the retained PCR edge fraction over 5%, 10%, 15%, 20% and 30%; (iii) graph construction between PCR and an absolute-Pearson graph at matched 15% density; and (iv) the knockout intervention among strict cut-in-and-out, cutting outgoing edges only, and removing incoming edges while redistributing the KO row uniformly over non-KO genes. For each configuration, we recomputed per-KO AUROC and AP and summarised the mean with a 95% percentile confidence interval from 5,000 fixed-seed bootstrap resamples of KO genes. Because the PageRank transition matrix must remain non-negative and row-stochastic, true signed-edge propagation was not included; the consequences of discarding regulatory sign are treated as a limitation rather than approximated with invalid negative transition probabilities. Results are reported in Supplementary Figure S6.

### 4.7 Statistics and reproducibility

We report predictive performance per KO and summarise across KOs using macro-averages. For the matched SERGIO comparison between TAKO and absolute correlation, KO genes are the paired units. We summarise paired differences with 95% percentile confidence intervals from 10,000 fixed-seed bootstrap resamples of KO genes and use a two-sided Wilcoxon signed-rank test as a descriptive paired analysis. Because the real-data evaluation contains only two distinct KO targets, we do not perform across-target significance testing and report Top- k results descriptively. For pathway enrichment analyses, we report FDR-adjusted p -values from preranked GSEA. Runtime was recorded for each KO and summed over the same prespecified KO lists used in the accuracy evaluation. For GenKI, one WT model-training time was added to the summed per-KO scoring time.

### 4.8 Implementation details

TAKO, the Pearson-correlation baselines, and the evaluation scripts were implemented in Python using NumPy (Harris et al., 2020) for numerical linear algebra, pandas for data manipulation (McKinney et al., 2010), and scikit-learn for evaluation metrics (Pedregosa et al., 2011). GenKI was run using its public Python implementation, whereas scTenifoldKnk was run using its public R implementation. For the primary TAKO analyses, we employed PCR-based graph construction with the following hyperparameters: H=3000 , D=50 , top-edge retention p=15% , ridge λ=0.05 , α=0.5 , iters = 200, tol = $10^{-8}$ . Regarding the specific perturbation and ranking strategies, we applied the strict cut-in-and-out intervention combined with uniform restart. Raw differential scoring was used for SERGIO and GSEA, and absolute differential scoring was used for comparison with unsigned measured-response magnitudes.

## Data Availability

This study analyses four previously released scRNA-seq datasets and simulated data generated using SERGIO. Specifically, datasets are available from GEO (GSE130626, part of GSE130627; and GSE129584, part of GSE129628), SRA (SRP135960), and the EGA under controlled access (EGAS00001004481). All dataset identifiers and accessions are provided above. Preprocessing steps, SERGIO simulation configurations and analysis settings are described in the Methods and Supplementary Material.
