# Supplementary material for: Topology-adjusted knockout (TAKO): interpretable in silico single-gene knockout ranking on wild-type single-cell gene regulatory networks
Source: Front Bioinform. 2026 Oct 14;6:1924703. doi: 10.3389/fbinf.2026.1924703 (PMC13714505; doi:10.3389/fbinf.2026.1924703)
Supplement: Supplementary file 1 [file DataSheet1.pdf]

Supplementary Information

for

Topology-Adjusted Knockout (TAKO): interpretable in silico single-gene knockout ranking on wild-type single-cell gene regulatory networks

Hao Wang[1] and Koji Oba[1,*]

[1]Graduate School of Interdisciplinary Information Studies, The University of Tokyo, Tokyo, Japan
[*]Correspondence: obakoji@iii.u-tokyo.ac.jp

This Supplementary Information includes: Supplementary Notes S1–S2, Supplementary Tables S1–S6, and Supplementary Figures S1–S7.

Contents

Supplementary Notes

Supplementary Note S1. Mathematical formalisation of TAKO as a topology-adjusted knockout framework . . . 2

Supplementary Note S2. Implementation details and algorithm . . . 4

Supplementary Tables

Supplementary Table S1. Summary of real scRNA-seq applications and selected gene lists . . . 6

Supplementary Table S2. Top-ranked genes in the microglia *Trem2* analysis . . . 7

Supplementary Table S3. Top-ranked genes in the AT1 *Nkx2-1* analysis . . . 8

Supplementary Table S4. Top-ranked genes in the epithelial *STAT1* analysis . . . 9

Supplementary Table S5. Top-ranked genes in the neuronal *Mecp2* analysis . . . 10

Supplementary Table S6. Pearson- and Spearman-correlation sensitivity analysis on SERGIO benchmarks . . . 11

Supplementary Figures

Supplementary Figure S1. Cell subsampling stability of TAKO . . . 12

Supplementary Figure S2. Relationship between expression, edge weight, and KO impact score 13

Supplementary Figure S3. Noise robustness of TAKO on SERGIO-400G . . . 14

Supplementary Figure S4. STRING network of top-ranked genes in the microglia *Trem2* analysis 15

Supplementary Figure S5. GO Biological Process enrichment of top-ranked genes in the microglia *Trem2* analysis . . . 16

Supplementary Figure S6. Sensitivity and ablation analyses on SERGIO-400G . . . 17

Supplementary Figure S7. Precision–recall curves for the SERGIO structural-reference benchmarks 18

Supplementary Note S1. Mathematical formalisation of TAKO as a topology-adjusted knockout framework

Setup. Let $W \in \mathbb{R}_{\geq 0}^{n\times n}$ be a non-negative gene–gene weight matrix constructed from WT scRNA-seq data using a principal component regression graph-inference pipeline. W_{ij} represents the directed edge weight from gene i to gene j. Let $d_i = \sum_{j=1}^{n} W_{ij}$ be the out-strength of node i and $D = \mathrm{diag}(d)$. We define a row-normalised matrix P by

$$P_{ij} = \begin{cases} \dfrac{W_{ij}}{d_i}, & d_i > 0, \\ 1, & d_i = 0 \text{ and } j = i, \\ 0, & d_i = 0 \text{ and } j \neq i, \end{cases} \tag{S1}$$

so every row sums to 1; a zero-outdegree node is assigned a self-loop, matching the implementation used in the reported analyses. Let $v \in \mathbb{R}_{\geq 0}^{n}$ be a restart vector with $\sum_j v_j = 1$ and let $\alpha \in (0, 1)$ be the propagation weight.

S1.1 Stationary propagation

We compute a stationary propagation profile $s \in \mathbb{R}_{\geq 0}^{n}$ as the fixed point of

$$s \;=\; (1-\alpha)\, v \;+\; \alpha\, sP, \tag{S2}$$

which is solved by power iteration in practice. Because P is non-negative and row-stochastic, its spectral radius is $\rho(P) = 1$. Therefore, for any $\alpha \in (0, 1)$, $(I - \alpha P)$ is invertible and the solution can be written as

$$s \;=\; (1-\alpha)\, v(I-\alpha P)^{-1} \;=\; (1-\alpha) \sum_{t=0}^{\infty} \alpha^t\, vP^t. \tag{S3}$$

S1.2 Topology-adjusted knockout as a strict cut-in-and-out intervention

For a KO gene g, TAKO constructs a topology-adjusted KO transition matrix P_g^{KO} using a strict cut-in-and-out intervention, implemented as a no-inflow/no-outflow edit. First, inflow to g is removed by clearing column g. Second, outflow from g is removed by replacing row g with a pure self-loop. Formally, define an edited matrix $\widetilde{P}_g^{\mathrm{KO}}$ by

$$\widetilde{P}_{g,ij}^{\mathrm{KO}} = \begin{cases} 1, & i = j = g \quad \text{self-loop at } g, \\ 0, & j = g,\ i \neq g \quad \text{ban inflow to } g, \\ 0, & i = g,\ j \neq g \quad \text{ban outflow from } g, \\ P_{ij}, & \text{otherwise.} \end{cases} \tag{S4}$$

We then apply a row re-normalisation step to obtain P_g^{KO}. Let $c_i = \sum_{j=1}^{n} \widetilde{P}_{g,ij}^{\mathrm{KO}}$. If $c_i = 0$, we assign a self-loop at i before normalisation. Hence,

$$P_{g,i:}^{\mathrm{KO}} = \begin{cases} \dfrac{\widetilde{P}_{g,i:}^{\mathrm{KO}}}{c_i}, & c_i > 0, \\ e_i^{\top}, & c_i = 0, \end{cases} \tag{S5}$$

where e_i is the i-th basis vector.

S1.3 KO impact as a stationary shift and derived scoring variants

Using the same restart vector v for WT and KO runs, we compute

$$s^{\mathrm{WT}} = (1-\alpha)v + \alpha s^{\mathrm{WT}} P, \qquad s_g^{\mathrm{KO}} = (1-\alpha)v + \alpha s_g^{\mathrm{KO}} P_g^{\mathrm{KO}}. \tag{S6}$$

The primary KO impact profile is the stationary shift

$$\Delta s_g^{\mathrm{raw}} \;=\; s^{\mathrm{WT}} - s_g^{\mathrm{KO}}. \tag{S7}$$

The implementation also supports two derived scoring variants:

$$\Delta s_g^{\mathrm{pos}} = \max(\Delta s_g^{\mathrm{raw}}, 0), \qquad \Delta s_g^{\mathrm{abs}} = \left|\Delta s_g^{\mathrm{raw}}\right|. \tag{S8}$$

For a target gene j, the corresponding KO impact scores are denoted by $\Delta s_{g,j}^{\mathrm{raw}}$, $\Delta s_{g,j}^{\mathrm{pos}}$, and $\Delta s_{g,j}^{\mathrm{abs}}$. The ranking score is analysis-specific: $\Delta s_{g,j}^{\mathrm{raw}}$ is used for the SERGIO structural benchmark and preranked GSEA, whereas $\Delta s_{g,j}^{\mathrm{abs}}$ is used for magnitude-based visualisation and for direct comparison with unsigned measured WT–KO response magnitudes.

S1.4 Numerical solution

In practice, TAKO solves (S2) by power iteration:

$$s^{(t+1)} \leftarrow (1-\alpha)v + \alpha s^{(t)} P, \tag{S9}$$

stopping when $\left\|s^{(t+1)} - s^{(t)}\right\|_1 < \texttt{tol}$ or at `iters`. The same procedure is applied to P_g^{KO} to obtain s_g^{KO}.

Supplementary Note S2. Implementation details and algorithm

Unless otherwise noted, preprocessing, graph construction, propagation, and evaluation settings are identical to those described in the main text (*System and methods*).

Algorithm S1 TAKO pipeline for WT-only single-gene KO impact ranking

Require: WT count matrix X (cells × genes), candidate KO gene index g, propagation weight α, tolerance `tol`, maximum iterations `iters`, and graph-construction parameters H, D, λ, τ

Ensure: KO impact profiles Δs_g^{raw}, Δs_g^{pos}, and Δs_g^{abs}, together with an analysis-specific ranked gene list

$X \leftarrow \text{CP10K\_LOG1P}(\text{SELECTHVG}(X; H) \cup \text{WHITELIST}(g))$
$W \leftarrow \text{INFERGRN\_PCR}(X; D, \lambda, \tau)$
$P \leftarrow \text{ROWNORM}(W)$
$v \leftarrow \text{RESTARTVECTOR}(g)$
$s^{\text{WT}} \leftarrow \text{PPR}(P, v, \alpha; \texttt{tol}, \texttt{iters})$
$\widetilde{P}_g^{\text{KO}} \leftarrow \text{CUTINANDOUTEDIT}(P, g)$
$P_g^{\text{KO}} \leftarrow \text{RENORMROWS}(\widetilde{P}_g^{\text{KO}})$
$s_g^{\text{KO}} \leftarrow \text{PPR}(P_g^{\text{KO}}, v, \alpha; \texttt{tol}, \texttt{iters})$
$\Delta s_g^{\text{raw}} \leftarrow s^{\text{WT}} - s_g^{\text{KO}}$
$\Delta s_g^{\text{pos}} \leftarrow \max(\Delta s_g^{\text{raw}}, 0)$
$\Delta s_g^{\text{abs}} \leftarrow |\Delta s_g^{\text{raw}}|$
Select $m \in \{\text{raw}, \text{pos}, \text{abs}\}$ according to the analysis objective
return Δs_g^{raw}, Δs_g^{pos}, Δs_g^{abs}, and $\text{RANK}(\Delta s_g^{m})$

Default configuration used in this study. Unless otherwise stated, we use PCR-based graph construction with $H = 3000$ highly variable genes, $D = 50$ principal components, ridge $\lambda = 0.05$, and sparsification $\tau = 15\%$, selecting the top 15% of off-diagonal entries by $|A|$. For propagation, we use $\alpha = 0.50$, `iters`=200, and `tol`=10^{-8}. We use a uniform restart vector over non-KO genes together with the strict cut-in-and-out intervention.

S2.1 Exact command-line configuration used for SERGIO benchmarks

All SERGIO benchmarks reported in this study use the same core TAKO settings:

```
--graph pcr
--hvg 3000
--genki-pc-d 50
--genki-top-pct 15
--genki-binarize 0
--genki-ridge 0.05
--alpha 0.50
--iters 200
--tol 1e-8
--restart uniform
--ko-mode no-in-out
--delta-mode raw
--topk 10,20,50
```

Dataset-specific inputs are the counts file, ground-truth GRN file, KO sequence file, and output directory (for 100G, 400G, and 1200G, respectively), while all core algorithmic parameters above are fixed. The KO

sequence files contain the prespecified genes with strict simulator-GRN out-degree > 10, yielding 6, 25, and 45 KO genes for the 100G, 400G, and 1200G benchmarks, respectively. For each KO gene, the KO gene itself is excluded from candidate targets, and simulator-defined direct outgoing neighbours are treated as structural-reference positives.

Computational complexity. If P is stored and multiplied as a sparse matrix with E non-zero entries, each PPR iteration costs $O(E)$. In the provided reference implementation, P is stored as a dense array; therefore, each iteration costs $O(n^2)$ in the worst case, while the effective sparsity is controlled by the masking step in graph construction.

S2.2 Precision–recall, sensitivity, and ablation analyses

SERGIO precision–recall curves. For each of the prespecified KO genes, we computed precision–recall curves using the same candidate genes and simulator-defined direct outgoing neighbours as in the AUROC and AP analyses. The displayed methods are TAKO with `delta-mode=raw`, signed Pearson correlation, absolute Pearson correlation, and a fixed-seed random ranking, all recalculated using the same KO targets and candidate genes. Per-KO curves were mapped to a common recall grid using a monotone precision envelope and then macro-averaged within each network size. Shaded bands show pointwise 95% percentile confidence intervals from 1,000 fixed-seed bootstrap resamples of KO genes, and horizontal dashed lines show the mean structural-positive prevalence.

Sensitivity and ablation analyses. We performed a one-factor-at-a-time analysis on the 400G SERGIO benchmark using the same 25 prespecified KO genes as in the primary comparison. Starting from the default configuration (PCR graph, 15% retained off-diagonal edges, $\alpha = 0.5$, and strict cut-in-and-out intervention), we varied: (i) propagation weight $\alpha \in \{0.1, 0.2, \ldots, 0.9\}$; (ii) retained PCR edge fraction over $\{5, 10, 15, 20, 30\}$%; (iii) graph construction between PCR and an absolute-Pearson graph at matched 15% density; and (iv) the KO intervention among strict cut-in-and-out, cutting outgoing edges only, and removing incoming edges while redistributing the KO row uniformly over non-KO genes. For each configuration, we recomputed per-KO AUROC and AP and report their means with 95% percentile confidence intervals from 5,000 fixed-seed bootstrap resamples of KO genes.

The transition matrix used by TAKO is non-negative and row-stochastic. Consequently, true signed-edge propagation was not included, because negative edge weights cannot be used directly as Markov transition probabilities. Rather than introducing an unvalidated signed-propagation surrogate, we state the loss of regulatory sign as a limitation of the current method.

Supplementary Table S1. Summary of real scRNA-seq applications and selected gene lists. For each dataset, we report the cell type of interest, target KO gene, data accession, and the selected genes used for downstream visualisation and enrichment analyses in the original WT-only application analyses (main-text Figs. 3–4). These application cell counts and gene lists are distinct from the measured WT–KO evaluation cohorts used in main-text Fig. 5.

Data set	Cell type of interest and number of cells	Target KO gene	Source	Selected genes
Microglia_Trem2	Microglia 648	*Trem2*	GEO: GSE130626 (part of GSE130627)	*Ctsd*, *Hexb*, *Cst3*, *C1qb*, *Tyrobp*, *Ctss*, *C1qc*, *Ctsz*, *C1qa*, *Ctsl*, *Laptm5*, *Lgmn*, *Psap*, *Ctsb*, *Ctsa*, *Lamp1*, *Fcer1g*, *Fcgr3*, *Itgb2*, *Itgam*, *Csf1r*, *Sirpa*, *Cd68*, *Cd53*, *Cd63*, *P2ry12*, *Tmem119*, *Abi3*, *Gpr34*, *Grn*
AT1_Nkx2-1	AT1 cells 624	*Nkx2-1*	GEO: GSE129584 (part of GSE129628)	*Aqp1*, *Aqp5*, *Ager*, *Cldn18*, *Cav1*, *Emp2*, *Cadm1*, *Esam*, *Alcam*, *Pgrmc1*, *Scnn1g*, *Slc1a5*, *Sparc*, *Fbln5*, *Npnt*, *Col4a3*, *Col4a4*, *Timp3*, *Mmp11*, *Serpinh1*, *Igfbp2*, *Msn*, *App*, *Tgm2*, *Mfge8*, *Matn4*, *Tspan8*, *Tspan15*, *Tspan13*, *Pdgfa*
COVID-19_STAT1	Epithelial cells 108	*STAT1*	EGAS000010004481	*CLDN7*, *KRT18*, *KRT19*, *ELF3*, *CD164*, *EZR*, *ACTN4*, *ARPC3*, *GSN*, *RAB11A*, *ARL1*, *VAMP8*, *CD63*, *YWHAZ*, *RAC1*, *ANXA2*, *CALR*, *PDIA3*, *POMP*, *TMEM165*, *TM9SF3*, *VMP1*, *LAMTOR5*, *UBB*, *UBC*, *TAP1*, *CYBA*, *CASP4*, *CTSB*, *CSTB*
Neurons_Mecp2	Neurons 2,054 and 2,156	*Mecp2*	SRA: SRP135960	*Map1b*, *Mapt*, *Stmn2*, *Kif1b*, *Dynll2*, *Dpysl2*, *Pafah1b1*, *Rtn3*, *Stxbp1*, *Vamp2*, *Syt4*, *Gap43*, *Ncam1*, *Uchl1*, *Ank2*, *Sptbn1*, *Gnb1*, *Gnao1*, *Gng2*, *Gnaq*, *Ywhab*, *Ywhae*, *Ywhaz*, *Ywhag*, *Rab6a*, *Gsk3b*, *Ppp2ca*, *Rufy3*, *Nsg2*, *Gdi1*

Supplementary Table S2. Top-ranked genes in the microglia *Trem2* analysis. Panel A shows the top 30 genes ranked by the signed KO impact score $\Delta s_{g,j}^{\text{raw}}$ with $\Delta s_{g,j}^{\text{raw}} > 0$, indicating that the WT stationary score exceeds the topology-adjusted KO stationary score. Panel B shows the top 30 genes ranked by the magnitude score $\Delta s_{g,j}^{\text{abs}} = |\Delta s_{g,j}^{\text{raw}}|$, with the sign of $\Delta s_{g,j}^{\text{raw}}$ retained. A negative value indicates that the KO stationary score exceeds the WT stationary score.

A. Top 30 by signed KO impact score. $\Delta s_{g,j}^{\text{raw}} > 0$

Rank	Gene	Type	Dir.	Δs
1	*CTSD*	PC	WT>KO	3.52×10^{-7}
2	*HEXB*	PC	WT>KO	3.13×10^{-7}
3	*CTSZ*	PC	WT>KO	2.93×10^{-7}
4	*CD9*	PC	WT>KO	2.84×10^{-7}
5	*TYROBP*	PC	WT>KO	2.66×10^{-7}
6	*SYNGR1*	PC	WT>KO	2.64×10^{-7}
7	*C1QC*	PC	WT>KO	2.58×10^{-7}
8	*CD68*	PC	WT>KO	2.56×10^{-7}
9	*TIMP2*	PC	WT>KO	2.54×10^{-7}
10	*CTSS*	PC	WT>KO	2.45×10^{-7}
11	*C1QB*	PC	WT>KO	2.44×10^{-7}
12	*CTSB*	PC	WT>KO	2.38×10^{-7}
13	*CST3*	PC	WT>KO	2.15×10^{-7}
14	*CYBA*	PC	WT>KO	2.14×10^{-7}
15	*CTSA*	PC	WT>KO	2.11×10^{-7}
16	*LAPTM5*	PC	WT>KO	2.03×10^{-7}
17	*OLFML3*	PC	WT>KO	2.01×10^{-7}
18	*CTSL*	PC	WT>KO	2.00×10^{-7}
19	*HEXA*	PC	WT>KO	2.00×10^{-7}
20	*C1QA*	PC	WT>KO	1.99×10^{-7}
21	*ABHD12*	PC	WT>KO	1.98×10^{-7}
22	*GRN*	PC	WT>KO	1.94×10^{-7}
23	*CREG1*	PC	WT>KO	1.82×10^{-7}
24	*RNASET2A*	PC	WT>KO	1.81×10^{-7}
25	*CST7*	PC	WT>KO	1.79×10^{-7}
26	*B2M*	PC	WT>KO	1.74×10^{-7}
27	*CD63*	PC	WT>KO	1.73×10^{-7}
28	*FCER1G*	PC	WT>KO	1.73×10^{-7}
29	*ITGB5*	PC	WT>KO	1.72×10^{-7}
30	*LY86*	PC	WT>KO	1.64×10^{-7}

B. Top 30 by magnitude score $\Delta s_{g,j}^{\text{abs}}$**, with sign retained**

Rank	Gene	Type	Dir.	Δs
1	*HIST1H2BC*	PC	KO>WT	-4.85×10^{-7}
2	*CCL3*	PC	KO>WT	-4.72×10^{-7}
3	*APOE*	PC	KO>WT	-4.49×10^{-7}
4	*HIST1H1C*	PC	KO>WT	-4.09×10^{-7}
5	*KLF2*	PC	KO>WT	-3.90×10^{-7}
6	*CTSD*	PC	WT>KO	3.52×10^{-7}
7	*DUSP1*	PC	KO>WT	-3.48×10^{-7}
8	*CCL4*	PC	KO>WT	-3.17×10^{-7}
9	*HEXB*	PC	WT>KO	3.13×10^{-7}
10	*SGK1*	PC	KO>WT	-2.94×10^{-7}
11	*CTSZ*	PC	WT>KO	2.93×10^{-7}
12	*CD9*	PC	WT>KO	2.84×10^{-7}
13	*H2-AB1*	PC	KO>WT	-2.69×10^{-7}
14	*TYROBP*	PC	WT>KO	2.66×10^{-7}
15	*SYNGR1*	PC	WT>KO	2.64×10^{-7}
16	*CITED2*	PC	KO>WT	-2.64×10^{-7}
17	*MT1*	PC	KO>WT	-2.61×10^{-7}
18	*TMEM176A*	PC	KO>WT	-2.59×10^{-7}
19	*C1QC*	PC	WT>KO	2.58×10^{-7}
20	*ALDOA*	PC	KO>WT	-2.57×10^{-7}
21	*CD68*	PC	WT>KO	2.56×10^{-7}
22	*TIMP2*	PC	WT>KO	2.54×10^{-7}
23	*HSPA5*	PC	KO>WT	-2.53×10^{-7}
24	*H2-AA*	PC	KO>WT	-2.52×10^{-7}
25	*ACTB*	PC	KO>WT	-2.52×10^{-7}
26	*TMSB10*	PC	KO>WT	-2.45×10^{-7}
27	*CTSS*	PC	WT>KO	2.45×10^{-7}
28	*BSG*	PC	KO>WT	-2.44×10^{-7}
29	*C1QB*	PC	WT>KO	2.44×10^{-7}
30	*FOS*	PC	KO>WT	-2.42×10^{-7}

Supplementary Table S3. Top-ranked genes in the AT1 *Nkx2-1* analysis. Panel A shows the top 30 genes ranked by the signed KO impact score $\Delta s^{\text{raw}}_{g,j}$ with $\Delta s^{\text{raw}}_{g,j} > 0$. Panel B shows the top 30 genes ranked by the magnitude score $\Delta s^{\text{abs}}_{g,j} = |\Delta s^{\text{raw}}_{g,j}|$, with the sign of $\Delta s^{\text{raw}}_{g,j}$ retained. Here $\Delta s^{\text{raw}}_{g,j} = s^{\text{WT}}_{g,j} - s^{\text{KO}}_{g,j}$ as defined in Eq. (S7). PC indicates protein-coding genes and lnc indicates long non-coding RNAs.

A. Top 30 by signed KO impact score. $\Delta s^{\text{raw}}_{g,j} > 0$

Rank	Gene	Type	Dir.	Δs
1	*Anxa8*	PC	WT>KO	2.54×10^{-7}
2	*Fuca1*	PC	WT>KO	2.35×10^{-7}
3	*Cxcl15*	PC	WT>KO	2.22×10^{-7}
4	*Plxnb2*	PC	WT>KO	2.14×10^{-7}
5	*Rtkn2*	PC	WT>KO	2.13×10^{-7}
6	*Cnot1*	PC	WT>KO	2.10×10^{-7}
7	*Pdlim1*	PC	WT>KO	2.03×10^{-7}
8	*Mrps6*	PC	WT>KO	1.99×10^{-7}
9	*Traf1*	PC	WT>KO	1.94×10^{-7}
10	*Vps35*	PC	WT>KO	1.88×10^{-7}
11	*Stard3*	PC	WT>KO	1.87×10^{-7}
12	*Cep78*	PC	WT>KO	1.87×10^{-7}
13	*Sftpd*	PC	WT>KO	1.82×10^{-7}
14	*Vsig2*	PC	WT>KO	1.77×10^{-7}
15	*Pcbd1*	PC	WT>KO	1.75×10^{-7}
16	*Fam114a2*	PC	WT>KO	1.75×10^{-7}
17	*Npdc1*	PC	WT>KO	1.72×10^{-7}
18	*Hint1*	PC	WT>KO	1.67×10^{-7}
19	*Sfn*	PC	WT>KO	1.64×10^{-7}
20	*Aqp5*	PC	WT>KO	1.62×10^{-7}
21	*Mvp*	PC	WT>KO	1.61×10^{-7}
22	*Faim*	PC	WT>KO	1.59×10^{-7}
23	*Arhgap31*	PC	WT>KO	1.59×10^{-7}
24	*Matn4*	PC	WT>KO	1.58×10^{-7}
25	*Atp6v1a*	PC	WT>KO	1.57×10^{-7}
26	*Ntf5*	PC	WT>KO	1.57×10^{-7}
27	*Rbpjl*	PC	WT>KO	1.57×10^{-7}
28	*Pop7*	PC	WT>KO	1.56×10^{-7}
29	*Abhd16a*	PC	WT>KO	1.55×10^{-7}
30	*Myh9*	PC	WT>KO	1.52×10^{-7}

B. Top 30 by magnitude score $\Delta s^{\text{abs}}_{g,j}$, with sign retained

Rank	Gene	Type	Dir.	Δs
1	*Meg3*	**lnc**	KO>WT	-1.96×10^{-6}
2	*Cyr61*	PC	KO>WT	-8.97×10^{-7}
3	*Malat1*	**lnc**	KO>WT	-8.51×10^{-7}
4	*Dusp1*	PC	KO>WT	-7.87×10^{-7}
5	*Igfbp2*	PC	KO>WT	-7.42×10^{-7}
6	*Fos*	PC	KO>WT	-7.41×10^{-7}
7	*Ccnd2*	PC	KO>WT	-7.38×10^{-7}
8	*Nnat*	PC	KO>WT	-7.38×10^{-7}
9	*Id1*	PC	KO>WT	-7.17×10^{-7}
10	*Crip1*	PC	KO>WT	-6.94×10^{-7}
11	*Actb*	PC	KO>WT	-6.53×10^{-7}
12	*Ifi27*	PC	KO>WT	-6.00×10^{-7}
13	*Btg2*	PC	KO>WT	-5.93×10^{-7}
14	*Neat1*	**lnc**	KO>WT	-5.93×10^{-7}
15	*Txnip*	PC	KO>WT	-5.87×10^{-7}
16	*Krt19*	PC	KO>WT	-5.82×10^{-7}
17	*Cldn3*	PC	KO>WT	-5.66×10^{-7}
18	*Xist*	**lnc**	KO>WT	-5.62×10^{-7}
19	*Calr*	PC	KO>WT	-5.60×10^{-7}
20	*Ier3*	PC	KO>WT	-5.53×10^{-7}
21	*Jun*	PC	KO>WT	-5.51×10^{-7}
22	*Tmem37*	PC	KO>WT	-5.36×10^{-7}
23	*Egr1*	PC	KO>WT	-5.35×10^{-7}
24	*Hspa5*	PC	KO>WT	-5.30×10^{-7}
25	*Tsc22d1*	PC	KO>WT	-5.28×10^{-7}
26	*Tubb4b*	PC	KO>WT	-5.22×10^{-7}
27	*Eef2*	PC	KO>WT	-5.22×10^{-7}
28	*Psap*	PC	KO>WT	-5.14×10^{-7}
29	*Serpinb9*	PC	KO>WT	-5.07×10^{-7}
30	*S100a10*	PC	KO>WT	-5.03×10^{-7}

Supplementary Table S4. Top-ranked genes in the epithelial *STAT1* analysis. Panel A shows the top 30 genes ranked by the signed KO impact score $\Delta s^{\mathrm{raw}}_{g,j}$ with $\Delta s^{\mathrm{raw}}_{g,j} > 0$. Panel B shows the top 30 genes ranked by the magnitude score $\Delta s^{\mathrm{abs}}_{g,j} = |\Delta s^{\mathrm{raw}}_{g,j}|$, with the sign of $\Delta s^{\mathrm{raw}}_{g,j}$ retained. Here $\Delta s^{\mathrm{raw}}_{g,j} = s^{\mathrm{WT}}_{g,j} - s^{\mathrm{KO}}_{g,j}$ as defined in Eq. (S7).

A. Top 30 by signed KO impact score. $\Delta s^{\mathrm{raw}}_{g,j} > 0$

Rank	Gene	Type	Dir.	Δs
1	*CD74*	PC	WT>KO	2.35×10^{-7}
2	*TAP1*	PC	WT>KO	2.23×10^{-7}
3	*POLR2G*	PC	WT>KO	2.13×10^{-7}
4	*RGCC*	PC	WT>KO	2.12×10^{-7}
5	*MARCH6*	PC	WT>KO	1.96×10^{-7}
6	*KRT13*	PC	WT>KO	1.89×10^{-7}
7	*MUC13*	PC	WT>KO	1.85×10^{-7}
8	*ATP2A2*	PC	WT>KO	1.79×10^{-7}
9	*CLEC2B*	PC	WT>KO	1.79×10^{-7}
10	*ANKLE2*	PC	WT>KO	1.76×10^{-7}
11	*LYN*	PC	WT>KO	1.74×10^{-7}
12	*STAT2*	PC	WT>KO	1.72×10^{-7}
13	*HLA-DRB1*	PC	WT>KO	1.71×10^{-7}
14	*EGR1*	PC	WT>KO	1.70×10^{-7}
15	*HES1*	PC	WT>KO	1.70×10^{-7}
16	*IL33*	PC	WT>KO	1.69×10^{-7}
17	*RIN2*	PC	WT>KO	1.63×10^{-7}
18	*SCG2*	PC	WT>KO	1.62×10^{-7}
19	*SF3B1*	PC	WT>KO	1.57×10^{-7}
20	*TRIM7*	PC	WT>KO	1.55×10^{-7}
21	*RAMP1*	PC	WT>KO	1.55×10^{-7}
22	*SUN1*	PC	WT>KO	1.53×10^{-7}
23	*IDO1*	PC	WT>KO	1.52×10^{-7}
24	*MTUS1*	PC	WT>KO	1.50×10^{-7}
25	*XIAP*	PC	WT>KO	1.49×10^{-7}
26	*SLC6A6*	PC	WT>KO	1.49×10^{-7}
27	*ADAM28*	PC	WT>KO	1.49×10^{-7}
28	*RSRC2*	PC	WT>KO	1.49×10^{-7}
29	*PCMTD1*	PC	WT>KO	1.46×10^{-7}
30	*SMIM5*	PC	WT>KO	1.44×10^{-7}

B. Top 30 by magnitude score $\Delta s^{\mathrm{abs}}_{g,j}$, with sign retained

Rank	Gene	Type	Dir.	Δs
1	*BPIFA1*	PC	KO>WT	-5.42×10^{-7}
2	*MALAT1*	lnc	KO>WT	-4.97×10^{-7}
3	*BPIFB1*	PC	KO>WT	-4.66×10^{-7}
4	*NEAT1*	lnc	KO>WT	-4.02×10^{-7}
5	*C15ORF48*	PC	KO>WT	-3.89×10^{-7}
6	*MT1E*	PC	KO>WT	-3.55×10^{-7}
7	*FABP5*	PC	KO>WT	-3.18×10^{-7}
8	*SPRR3*	PC	KO>WT	-3.06×10^{-7}
9	*S100A9*	PC	KO>WT	-3.03×10^{-7}
10	*ADIRF*	PC	KO>WT	-3.01×10^{-7}
11	*MSMB*	PC	KO>WT	-2.94×10^{-7}
12	*CSTA*	PC	KO>WT	-2.93×10^{-7}
13	*PIGR*	PC	KO>WT	-2.93×10^{-7}
14	*MGST1*	PC	KO>WT	-2.88×10^{-7}
15	*ATP1A1*	PC	KO>WT	-2.84×10^{-7}
16	*LCN2*	PC	KO>WT	-2.76×10^{-7}
17	*LY6D*	PC	KO>WT	-2.75×10^{-7}
18	*VMO1*	PC	KO>WT	-2.71×10^{-7}
19	*PERP*	PC	KO>WT	-2.71×10^{-7}
20	*SERPINB3*	PC	KO>WT	-2.69×10^{-7}
21	*AQP5*	PC	KO>WT	-2.68×10^{-7}
22	*RARRES1*	PC	KO>WT	-2.66×10^{-7}
23	*GDF15*	PC	KO>WT	-2.62×10^{-7}
24	*ANXA1*	PC	KO>WT	-2.58×10^{-7}
25	*S100A14*	PC	KO>WT	-2.56×10^{-7}
26	*TACSTD2*	PC	KO>WT	-2.54×10^{-7}
27	*MT1X*	PC	KO>WT	-2.51×10^{-7}
28	*IFI6*	PC	KO>WT	-2.51×10^{-7}
29	*AGR2*	PC	KO>WT	-2.49×10^{-7}
30	*PI3*	PC	KO>WT	-2.48×10^{-7}

Supplementary Table S5. Top-ranked genes in the neuronal *Mecp2* analysis. Panel A shows the top 30 genes ranked by the signed KO impact score $\Delta s_{g,j}^{\text{raw}}$ with $\Delta s_{g,j}^{\text{raw}} > 0$. Panel B shows the top 30 genes ranked by the magnitude score $\Delta s_{g,j}^{\text{abs}} = |\Delta s_{g,j}^{\text{raw}}|$, with the sign of $\Delta s_{g,j}^{\text{raw}}$ retained. Here $\Delta s_{g,j}^{\text{raw}} = s_{g,j}^{\text{WT}} - s_{g,j}^{\text{KO}}$ as defined in Eq. (S7).

A. Top 30 by signed KO impact score. $\Delta s_{g,j}^{\text{raw}} > 0$

Rank	Gene	Type	Dir.	Δs
1	*CYFIP2*	PC	WT>KO	1.82×10^{-7}
2	*GABRB3*	PC	WT>KO	1.55×10^{-7}
3	*HNRNPA2B1*	PC	WT>KO	1.54×10^{-7}
4	*SPTBN1*	PC	WT>KO	1.52×10^{-7}
5	*CLTA*	PC	WT>KO	1.38×10^{-7}
6	*SETD3*	PC	WT>KO	1.32×10^{-7}
7	*AATK*	PC	WT>KO	1.29×10^{-7}
8	*IGF1R*	PC	WT>KO	1.28×10^{-7}
9	*GLRB*	PC	WT>KO	1.27×10^{-7}
10	*KRIT1*	PC	WT>KO	1.27×10^{-7}
11	*ATP5J2*	PC	WT>KO	1.25×10^{-7}
12	*GPR162*	PC	WT>KO	1.19×10^{-7}
13	*SPTAN1*	PC	WT>KO	1.17×10^{-7}
14	*RCAN2*	PC	WT>KO	1.16×10^{-7}
15	*ZDHHC21*	PC	WT>KO	1.15×10^{-7}
16	*FAM219A*	PC	WT>KO	1.15×10^{-7}
17	*NEDD4*	PC	WT>KO	1.14×10^{-7}
18	*CNTNAP5A*	PC	WT>KO	1.13×10^{-7}
19	*NDUFC1*	PC	WT>KO	1.12×10^{-7}
20	*SBNO1*	PC	WT>KO	1.09×10^{-7}
21	*UBE4B*	PC	WT>KO	1.09×10^{-7}
22	*FXYD6*	PC	WT>KO	1.09×10^{-7}
23	*GPM6B*	PC	WT>KO	1.09×10^{-7}
24	*PCBP2*	PC	WT>KO	1.08×10^{-7}
25	*SRSF1*	PC	WT>KO	1.07×10^{-7}
26	*CHCHD10*	PC	WT>KO	1.06×10^{-7}
27	*STX4A*	PC	WT>KO	1.05×10^{-7}
28	*VAT1L*	PC	WT>KO	1.04×10^{-7}
29	*RPL37*	PC	WT>KO	1.04×10^{-7}
30	*ARHGEF9*	PC	WT>KO	1.03×10^{-7}

B. Top 30 by magnitude score $\Delta s_{g,j}^{\text{abs}}$, with sign retained

Rank	Gene	Type	Dir.	Δs
1	*CYFIP2*	PC	WT>KO	1.82×10^{-7}
2	*GABRB3*	PC	WT>KO	1.55×10^{-7}
3	*HNRNPA2B1*	PC	WT>KO	1.54×10^{-7}
4	*SPTBN1*	PC	WT>KO	1.52×10^{-7}
5	*CLTA*	PC	WT>KO	1.38×10^{-7}
6	*SETD3*	PC	WT>KO	1.32×10^{-7}
7	*AATK*	PC	WT>KO	1.29×10^{-7}
8	*IGF1R*	PC	WT>KO	1.28×10^{-7}
9	*KRIT1*	PC	WT>KO	1.27×10^{-7}
10	*GLRB*	PC	WT>KO	1.27×10^{-7}
11	*ATP5J2*	PC	WT>KO	1.25×10^{-7}
12	*GPR162*	PC	WT>KO	1.19×10^{-7}
13	*SPTAN1*	PC	WT>KO	1.17×10^{-7}
14	*RCAN2*	PC	WT>KO	1.16×10^{-7}
15	*ZDHHC21*	PC	WT>KO	1.15×10^{-7}
16	*FAM219A*	PC	WT>KO	1.15×10^{-7}
17	*NEDD4*	PC	WT>KO	1.14×10^{-7}
18	*CNTNAP5A*	PC	WT>KO	1.13×10^{-7}
19	*NDUFC1*	PC	WT>KO	1.12×10^{-7}
20	*GPM6B*	PC	WT>KO	1.09×10^{-7}
21	*SBNO1*	PC	WT>KO	1.09×10^{-7}
22	*FXYD6*	PC	WT>KO	1.09×10^{-7}
23	*UBE4B*	PC	WT>KO	1.09×10^{-7}
24	*PCBP2*	PC	WT>KO	1.08×10^{-7}
25	*SRSF1*	PC	WT>KO	1.07×10^{-7}
26	*CHCHD10*	PC	WT>KO	1.06×10^{-7}
27	*STX4A*	PC	WT>KO	1.05×10^{-7}
28	*RPL37*	PC	WT>KO	1.04×10^{-7}
29	*VAT1L*	PC	WT>KO	1.04×10^{-7}
30	*PI4KA*	PC	WT>KO	1.03×10^{-7}

Supplementary Table S6. Pearson- and Spearman-correlation sensitivity analysis on the SERGIO structural-reference benchmarks. For each network, entries report mean AUROC / mean average precision (AP), macro-averaged across the same prespecified KO genes used in the primary comparison (6, 25, and 45 KO genes for the 100G, 400G, and 1,200G networks, respectively). All correlation baselines use exactly the same WT cells, candidate genes, KO targets, and simulator-defined direct-outgoing-neighbour labels as TAKO.

Network	Signed Pearson r	Absolute Pearson $\|r\|$	Signed Spearman ρ	Absolute Spearman $\|\rho\|$
100 genes	0.613 / 0.571	0.630 / 0.624	0.615 / 0.570	0.618 / 0.600
400 genes	0.518 / 0.346	0.816 / 0.543	0.520 / 0.356	0.819 / 0.555
1,200 genes	0.513 / 0.340	0.897 / 0.580	0.514 / 0.338	0.897 / 0.580

Note. Pearson coefficients were computed directly from log-normalised WT expression values. Spearman coefficients were computed after replacing each gene's WT expression values by their ranks, using average ranks for ties. Signed baselines rank candidates by r or ρ, whereas absolute baselines rank candidates by $|r|$ or $|\rho|$. The close agreement between the absolute Pearson and absolute Spearman results shows that the strong absolute-correlation baseline is not specific to Pearson's linear scale.

Supplementary Fig. S1. Cell subsampling stability of TAKO. For each subsampling fraction, KO impact scores are computed from 10 random cell subsamples and compared with the full-data reference. Panel A shows top-k overlap, reported as the Jaccard index for $k \in \{30, 50\}$. Panel B shows rank stability, reported as the Spearman correlation between KO impact score vectors.

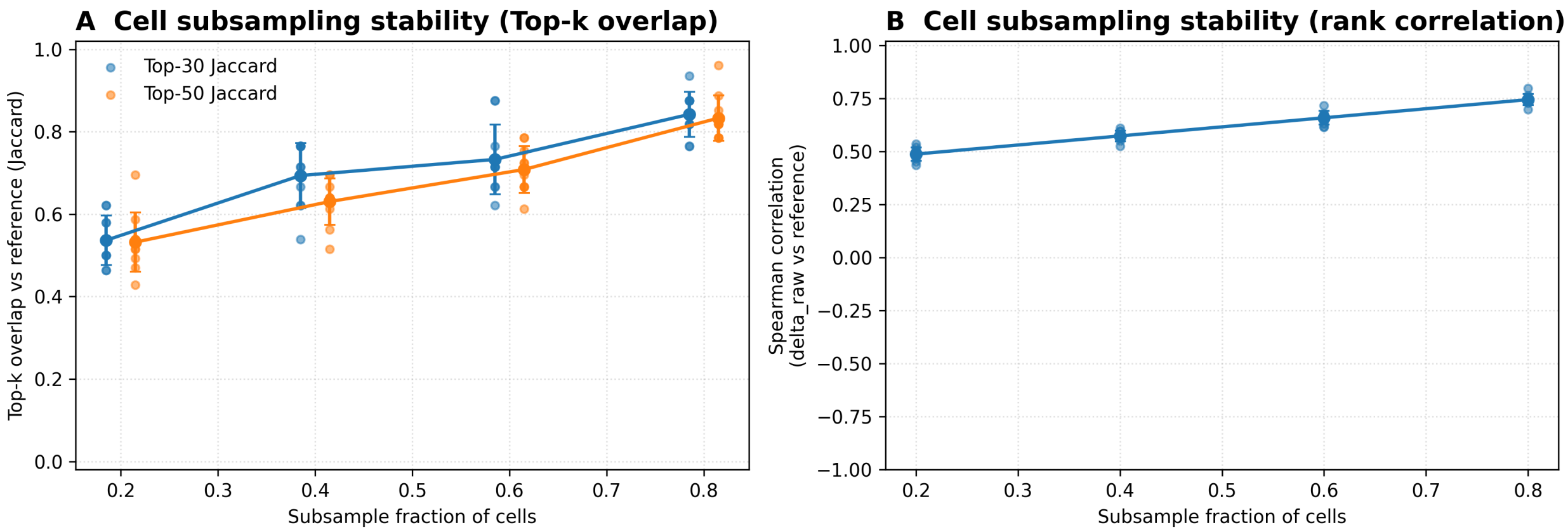

Supplementary Fig. S2. Relationship between gene expression, edge weight, and KO impact score in the microglia *Trem2* analysis. Panel A shows mean gene expression measured on the CP10K-log1p scale versus the signed KO impact score $\Delta s^{\text{raw}}_{g,j}$. Panel B shows WT scGRN edge weights versus $\Delta s^{\text{raw}}_{g,j}$. Zero-weight edges are jittered along the x-axis for visualisation. Highlighted genes are selected by the magnitude score $\Delta s^{\text{abs}}_{g,j} = |\Delta s^{\text{raw}}_{g,j}|$.

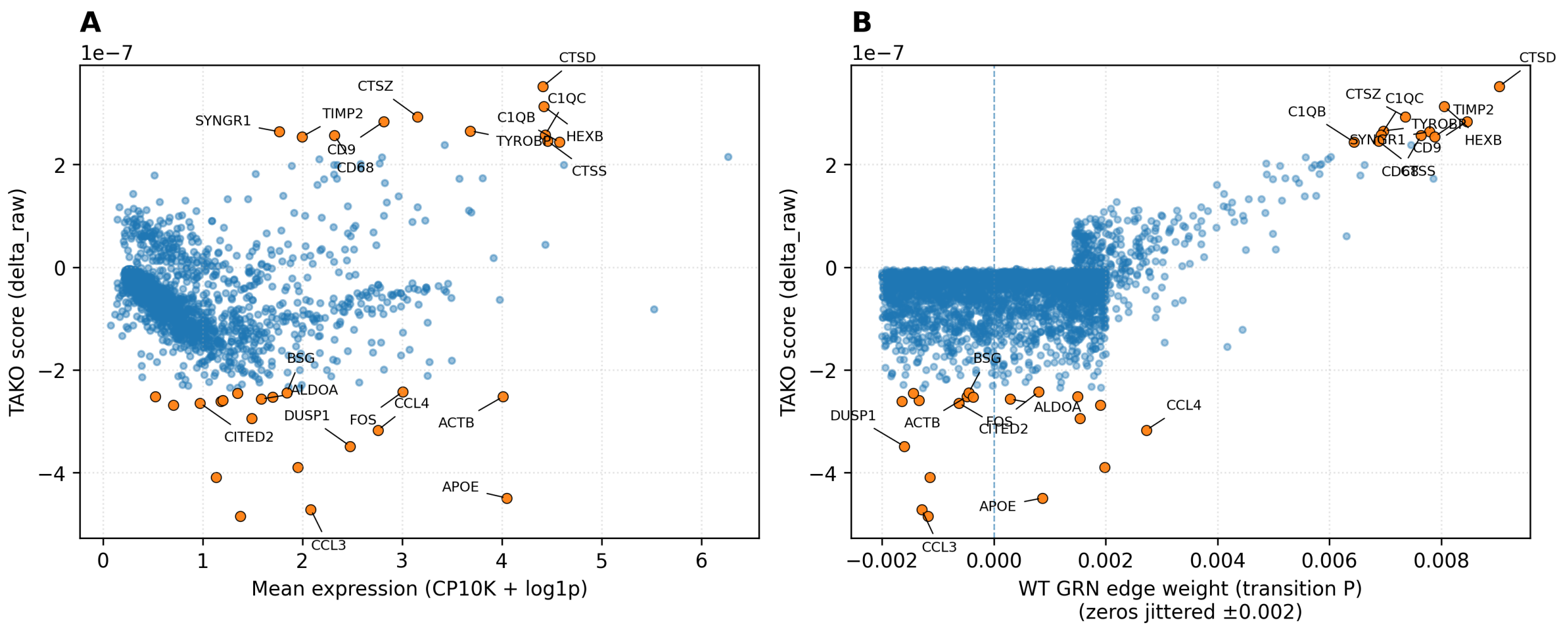

Supplementary Fig. S3. Noise robustness of TAKO on SERGIO-400G. Gaussian noise with standard deviation $\sigma \in \{0, 0.5, 1, 2\}$ is added to the CP10K-log1p expression matrix, and the full TAKO pipeline is re-run for 10 repeats per noise level. Panel A shows the distribution of KO-ranking performance measured by average precision across repeats. Panel B shows the distribution of KO-ranking performance measured by AUROC across repeats. Dashed lines indicate the baseline median at $\sigma = 0$.

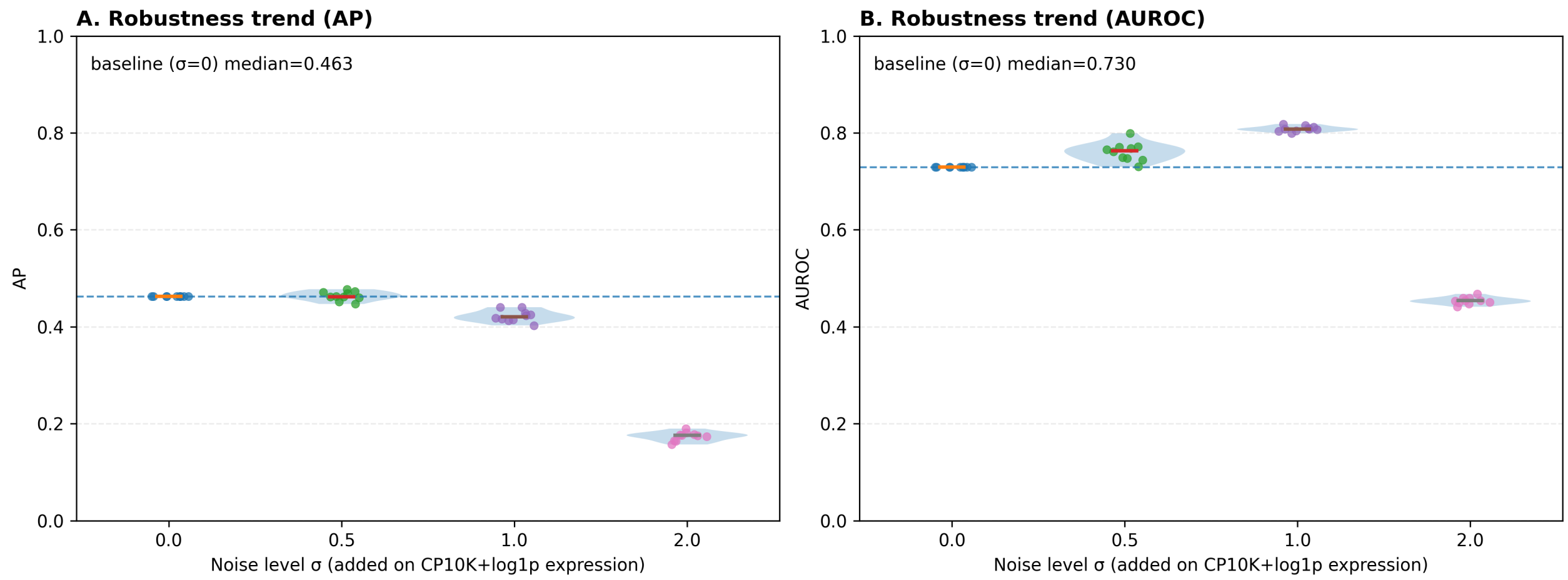

Supplementary Fig. S4. STRING network of top-ranked genes in the microglia *Trem2* analysis. Genes were selected by the magnitude score $\Delta s_{g,j}^{\mathrm{abs}}$ and visualised in STRING. Network modules were identified using MCL clustering with inflation parameter 3.

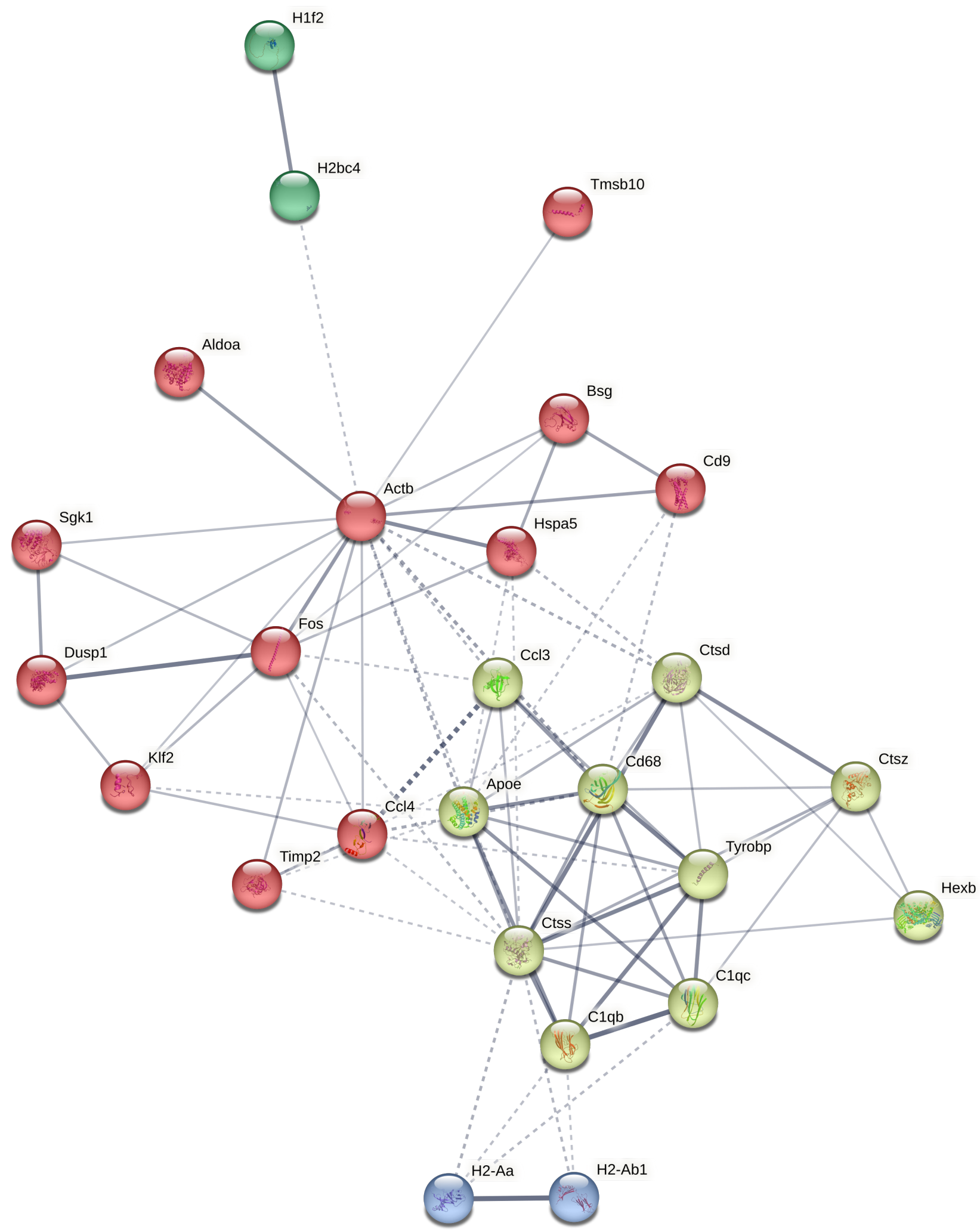

Supplementary Fig. S5. GO Biological Process enrichment of top-ranked genes in the microglia *Trem2* analysis. Enrichment analysis was performed on genes selected by the magnitude score $\Delta s^{\mathrm{abs}}_{g,j}$. The plot shows the top terms grouped by semantic similarity of at least 0.8 and ranked by $-\log_{10}(\mathrm{FDR})$. Bubble size indicates gene count.

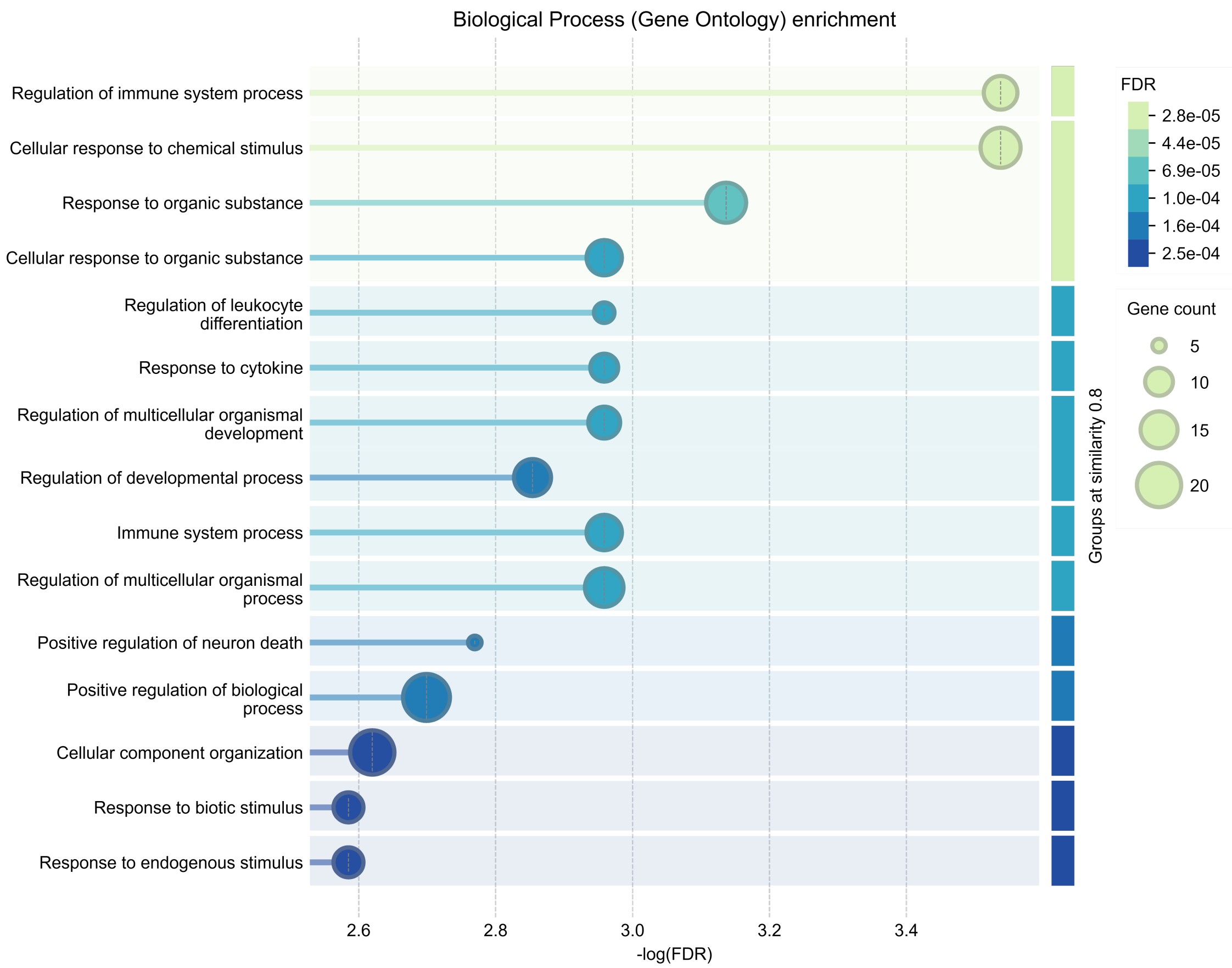

Supplementary Fig. S6. Sensitivity and ablation analyses on SERGIO-400G. All panels use the same 25 prespecified KO genes and report mean AUROC (orange circles) and mean average precision (blue squares); error bars are 95% percentile confidence intervals from 5,000 fixed-seed bootstrap resamples of KO genes. **A**, propagation weight α from 0.1 to 0.9. **B**, retained off-diagonal PCR edge fraction from 5% to 30%. **C**, PCR versus an absolute-Pearson graph at matched 15% density. **D**, strict cut-in-and-out, cutting outgoing edges only, and removing incoming edges while redistributing the KO row uniformly over non-KO genes. Grey highlighting or vertical dashed lines identify the default configuration: PCR, 15% retained edges, $\alpha = 0.5$, and strict cut-in-and-out intervention.

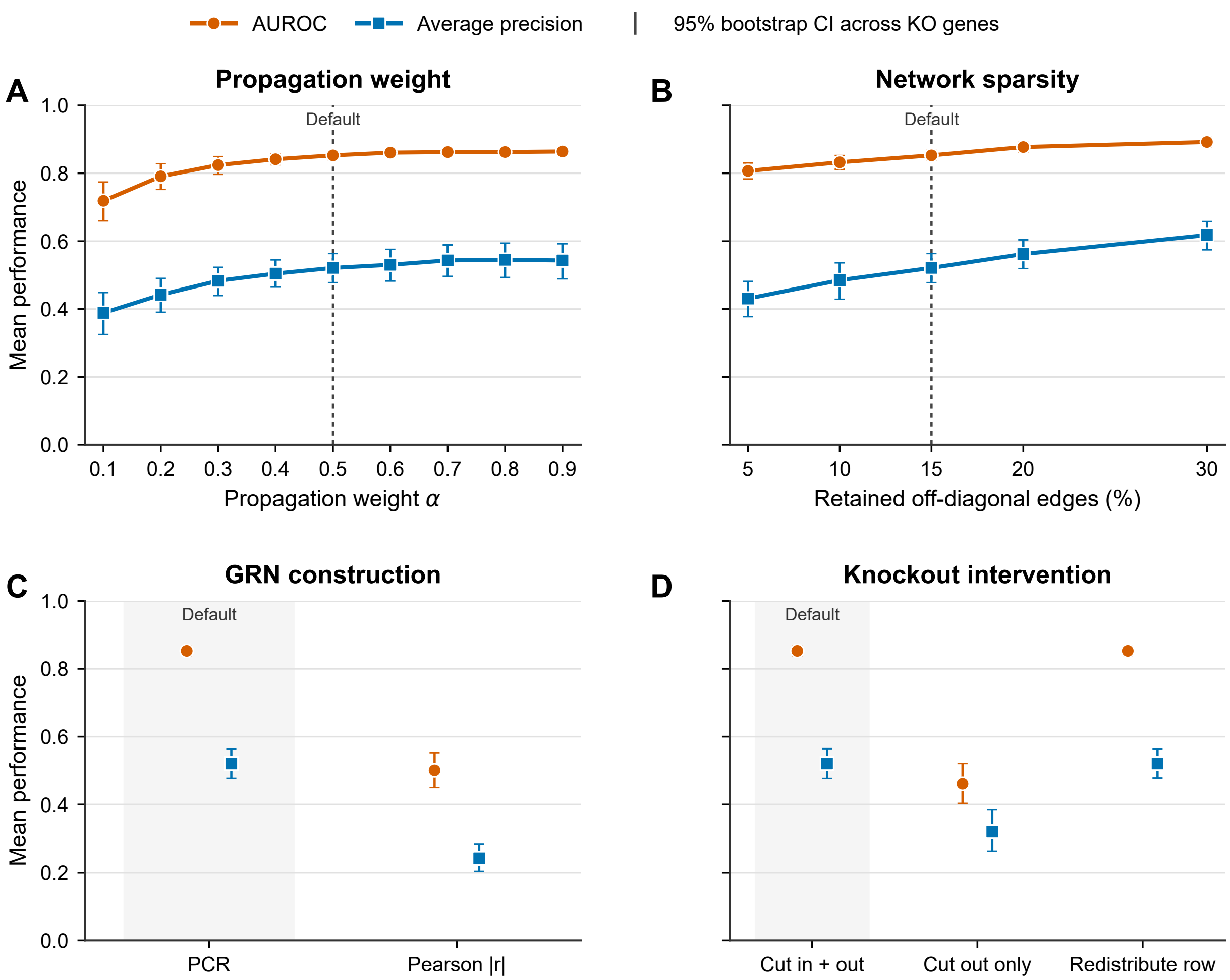

Supplementary Fig. S7. Precision–recall curves for the SERGIO structural-reference benchmarks. A–C, macro-averaged precision–recall curves for the 100G, 400G, and 1,200G networks, using 6, 25, and 45 prespecified KO genes, respectively. Positive labels are simulator-defined direct outgoing neighbours, so these panels evaluate structural-neighbour recovery rather than the complete transcriptional response after experimental KO. Curves are shown for TAKO with raw differential scoring, absolute Pearson correlation, signed Pearson correlation, and a fixed-seed random ranking. Shaded bands are pointwise 95% bootstrap confidence intervals across KO genes; horizontal dashed lines indicate mean structural-positive prevalence. The KO gene itself is excluded from candidate targets.

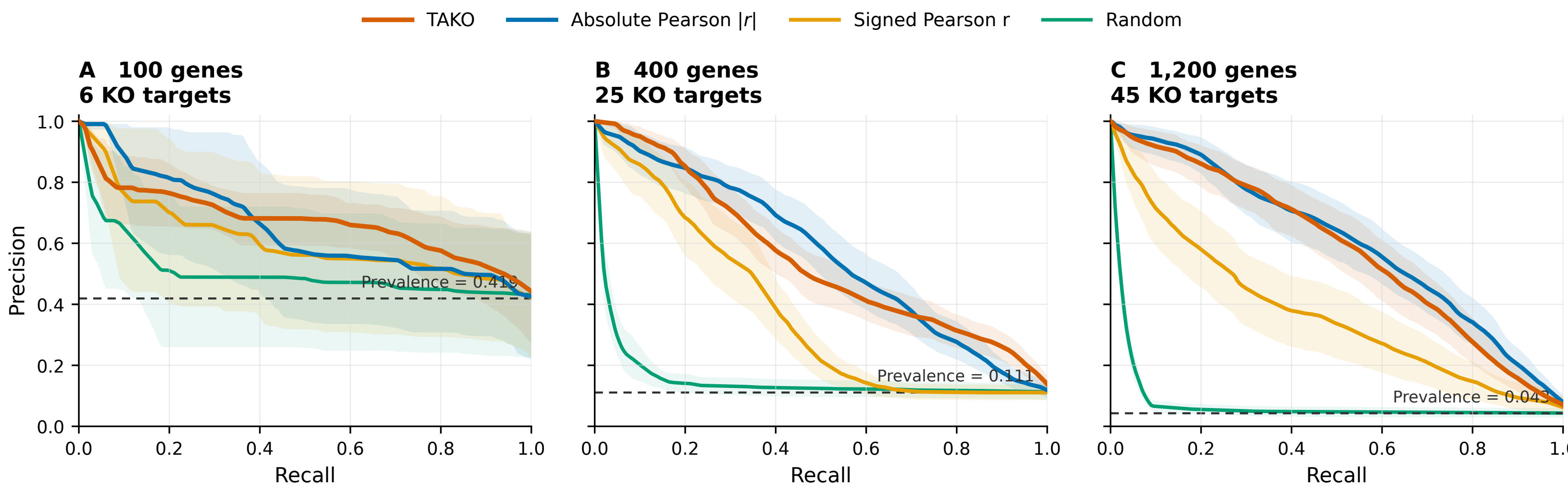

Supplementary Source Data

Source data underlying Supplementary Figures S1–S7 and Supplementary Tables S1–S6 are provided in the accompanying revision source-data files where applicable. Dataset identifiers, preprocessing settings, SERGIO configurations, and analysis scripts required for reproduction are described in the main text, this Supplementary Information, and the public TAKO repository.
